# Toward an Integrated Strategy for Volumetric Muscle Loss Regeneration

**DOI:** 10.3390/jcm15155901

**Published:** 2026-07-28

**Authors:** Christopher D’Costa, Kevin L. Zhang, Matthew Duazo, Vladimir Grubišić, Rabab Hamzah, Karrer Alghazali

**Affiliations:** 1 Department of Electrical and Computer Engineering, New York Institute of Technology, New York, NY 10023, USA; 2 Tissue Engineering and Advanced Materials (TEAM) Laboratory, Department of Electrical and Computer Engineering, College of Engineering and Computing Sciences, New York Institute of Technology, Old Westbury, NY 11568, USA; 3 College of Osteopathic Medicine, New York Institute of Technology, Old Westbury, NY 11568, USA; 4 Department of Biomedical Sciences, College of Osteopathic Medicine, New York Institute of Technology, Old Westbury, NY 11568, USA; 5 Center for Biomedical Innovation, College of Osteopathic Medicine, New York Institute of Technology, Old Westbury, NY 11568, USA; 6 Department of Biological and Chemical Sciences, New York Institute of Technology, Old Westbury, NY 11568, USA

**Keywords:** clinically translatable, regenerative medicine, volumetric muscle loss, tissue engineering, 3D bioprinting, exosome therapy

## Abstract

Volumetric muscle loss (VML) constitutes a significant clinical challenge, defined by the irreversible loss of skeletal muscle tissue and resulting in persistent functional deficits due to fibrosis, chronic inflammation, and insufficient endogenous regeneration. Existing clinical interventions, such as autologous grafting and free functional muscle transfer, are constrained by donor-site morbidity including infection, pain, suboptimal vascularization, and limited functional integration. Although tissue engineering has advanced considerably, no FDA-approved regenerative therapies currently exist for VML, underscoring a substantial translational gap. This review provides a systems-level synthesis of skeletal muscle repair through integrating fundamental biological processes, such as inflammation, satellite-cell activation, myogenesis, angiogenesis, and neuromuscular junction formation, with recent advances in biomaterials, scaffold engineering, and biofabrication technologies. The analysis addresses how critical scaffold design parameters, including alignment, porosity, stiffness, degradation kinetics, and bioactivity, influence cellular responses and tissue integration. Additionally, emerging strategies such as 3D bioprinting, nanofiber-based architectures, stem cell and exosome therapies, and bio-functional stimulation are evaluated inside a unified mechanobiological framework. This analysis is further extended to the regulatory setting, with emphasis on how scaffold composition, mechanism of action, and degree of biological integration affect classification pathways governed by the U.S. Food and Drug Administration. Most advanced VML therapies are anticipated to be regulated as combination products, which will require rigorous preclinical validation, standardized manufacturing processes, and carefully designed clinical studies. By integrating biological principles, engineering design, and regulatory considerations, this review highlights key opportunities, remaining challenges, and future priorities for the clinical translation of next-generation regenerative strategies for VML.

## 1. Overview

This review offers a structured analysis of regenerative strategies for volumetric muscle loss (VML), focusing on the integration of biological mechanisms, biomaterial design, biofabrication technologies, and translational factors to restore functional skeletal muscle. The literature was identified through searches of PubMed, Scopus, Web of Science, and Google Scholar, focusing primarily on publications from 2010 to 2026, and is organized into seven thematic sections of this review:

1. Skeletal Muscle Biology and Current Therapeutic Strategies for VML;
2. Design Principles and Emerging Strategies in Scaffold-Based Muscle Regeneration;
3. Cellular and Exosome-Based Therapies;
4. Bio-functional Muscle Stimulation Approaches;
5. Vascularization and Innervation as Critical Requirements for Functional Integration;
6. Immune Modulation as a Therapeutic Strategy in VML; and
7. Regulatory Consideration and FDA Approval Pathways for VML Scaffold Platform Therapeutics.

## 2. Introduction

Volumetric muscle loss (VML) is a debilitating musculoskeletal injury that causes profound long-term functional impairment, leading to potentially billions of dollars in annual healthcare costs in the United States when considering reconstructive intervention, rehabilitative treatment, and long-term disability management [1]. In essence, VML occurs when the extent of muscle damage exceeds the muscle’s intrinsic ability to repair and regenerate. Skeletal muscle is a highly plastic tissue with endogenous regenerative capacity following minor injuries. The activation of resident muscle stem cells (MuSCs), often referred to as satellite cells, allows for the proliferation and differentiation of myoblasts to myocytes, which fuse and repair damaged myofibers [2]. However, this intrinsic mechanism becomes overwhelmed when the stromal scaffolding, consisting of basal lamina and interstitial extracellular matrix (ECM), is critically ablated along with the parenchymal cells, which typically occurs when an injury exceeds a threshold of muscle volume, approximately 20% [3]. Consequently, the repair and regeneration process is supplanted by chronic inflammation, tissue degeneration, irreversible fibrosis, and, in severe cases, limb dysfunction or necrosis, requiring amputation.

VML arises from diverse etiologies, including trauma, combat, and surgery. In the military context, improvised explosive device (IED) blasts represent the predominant mechanism, with lower extremity injuries characterized by extensive soft-tissue cavitation [4,5]. Corona et al. reported that muscle injuries accounted for 65% of combat-related conditions in a cohort of discharged military personnel, with over 90% of diagnoses being identified as VML [6]. In the civilian context, vehicular trauma, industrial accidents, and tumor resection constitute common causes of VML.

**Figure 1:**
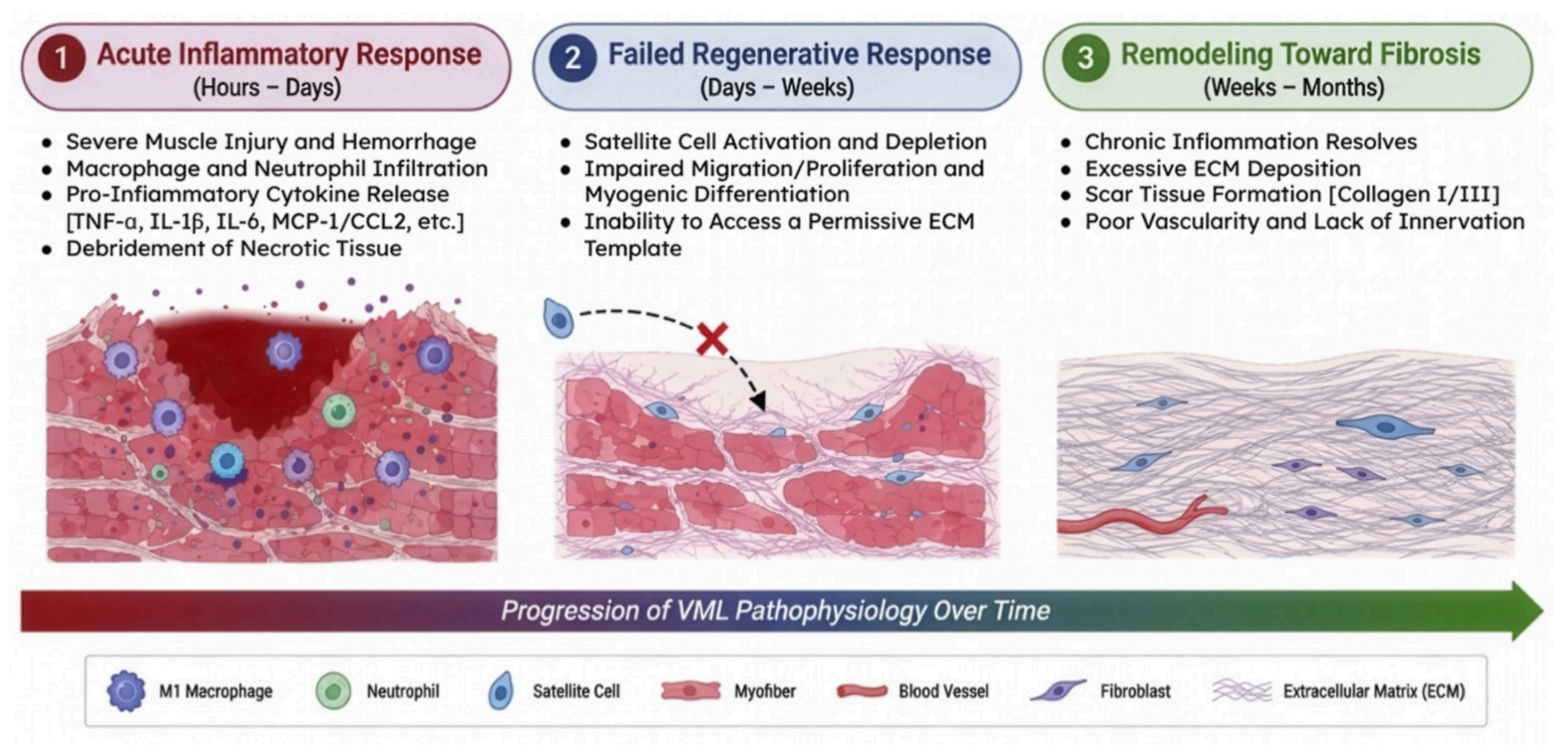
Phases of volumetric muscle loss (VML) pathophysiology and progression toward fibrotic, non-functional tissue. “This figure was generated using the AI-assisted design tool FigureLabs (web version, accessed on 1 April 2026; https://figurelabs.ai/). The initial layouts were subsequently refined, labeled, and verified by the authors to ensure scientific accuracy”.

The pathophysiology of VML is characterized by three overlapping phases (Figure 1): an initial acute inflammatory phase dominated by macrophage infiltration and pro-inflammatory signaling, a failed regenerative response phase involving the depletion of satellite cells and deterioration of the ECM, and a dysregulated remodeling phase which resolves toward fibrotic scar tissue rather than organized myofiber architecture [7,8]. Consequently, the resulting tissue is neuromuscularly inactive, devoid of functional motor innervation and thus incapable of generating the contractile and tensile forces required for normal muscle function.

Over the past decade, tissue engineering and regenerative medicine have emerged as the principal scientific framework for addressing and treating VML. The fundamental premise is to provide a decellularized or cell-integrated scaffold that recapitulates key biophysical and biochemical aspects of native muscle ECM, including spatial anisotropy, ligand adhesion, macrophage polarization, and vascular channels, in order to guide cell infiltration, myogenic differentiation, reinnervation, vascularization, and ultimately the reconstruction of functional contractile muscle [9,10]. The rapid advances in biomaterial science, bioelectronics, nanotechnology, and cell biology have produced a broad yet fragmented literature, making it difficult for researchers and clinicians to integrate the field’s current state as well as identify key unresolved challenges.

This review aims to fill the gap by providing a comprehensive synthesis of skeletal muscle regeneration strategies, utilizing literature from 2006 to 2026 in order to integrate pathophysiology, biomaterial design, and therapeutic approaches within a unified framework. We analyze current clinical approaches and their limitations, trace the evolution of scaffold design principles, examine the contributions of stem cell biology and exosomes, review the strategies for biophysical stimulation, assess the requirements for vascularization, discuss the emerging role of immune modulation, and finally, map the regulatory pathways governing the translation of these combinatorial platforms into clinical products. We conclude by proposing an integrated framework for VML, which coordinates these strategies temporally and spatially to maximize the probability of functional recovery. We conclude by proposing an integrated, staged framework for VML treatment that coordinates these strategies temporally and spatially to maximize the probability of durable functional recovery.

## 3. Skeletal Muscle Biology and Current Therapeutic Strategies for VML

The current clinical standard of care for VML has evolved incrementally from traditional reconstructive surgery and remains fundamentally constrained by the reliance on tissue transfer and rehabilitation. A thorough understanding of the limitations of these current treatments provides the essential context for recognizing emerging regenerative technologies as a fundamental paradigm shift rather than an incremental advancement in the management and treatment of VML.

### 3.1. Skeletal Muscle Architecture

**Figure 2:**
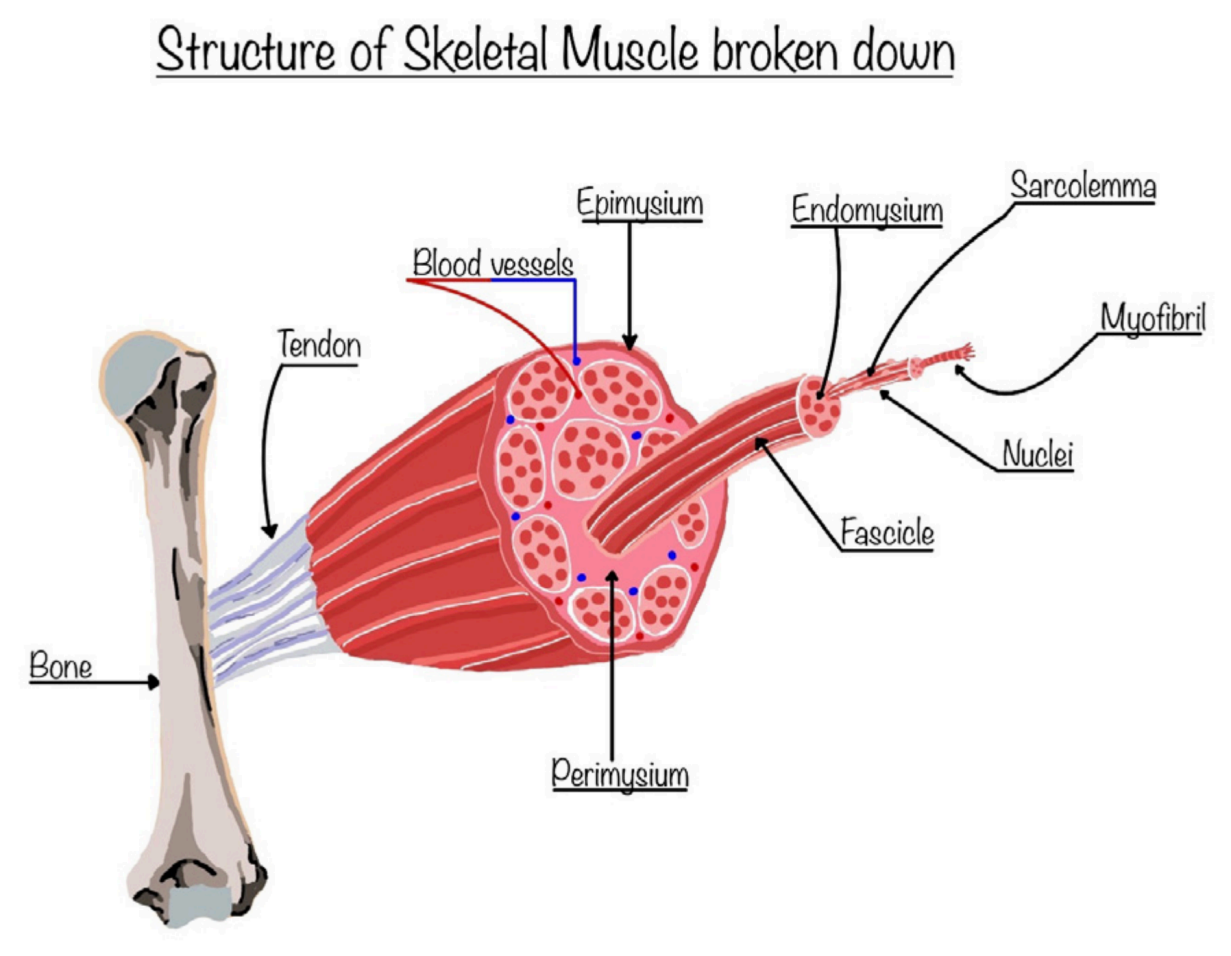
Skeletal muscle structure overview.

Before discussing these limitations, it is important to establish the complex structural and functional organization of native skeletal muscle. Skeletal muscle is a highly specialized, hierarchically organized tissue composed of multinucleated muscle cells, often referred to as muscle fibers [11]. These muscle fibers are composed of thousands of sarcomeres, the fundamental contractile unit, composed of thin actin filaments interdigitating between thick myosin filaments. These fibers are organized into muscle fascicles, which themselves are organized into the whole muscle, with each being protected by three distinct layers of connective tissue that comprise the ECM: the endomysium which protects individual fibers, the perimysium which protects individual fascicles, and the epimysium which protects the whole muscle (Figure 2).

Beyond its function in providing structural support, the ECM serves as a dynamic biochemical and biomechanical interface, regulating cell adhesion and migration, mechanotransduction, and force transmission, while maintaining the satellite microenvironment, which is essential for muscle repair and regeneration.

An extensive array of vascular and neural networks is equally critical to skeletal muscle function. A dense capillary bed supplies oxygen and nutrients, while removing metabolic waste products, supporting the high energetic demands of consecutive muscle contractions [12]. Angiogenesis is also vital for tissue development, maintenance, and regeneration. Muscle contraction is mediated by motor efferents, as α-motor neurons form neuromuscular junctions with individual myofibers to transmit action potentials that initiate excitation-contraction coupling [13]. Sensory afferents, such as Golgi tendon organs and muscle spindles, provide continuous proprioceptive feedback necessary for coordinated motor control [14]. Thus, the functional integrity of skeletal muscle depends not only on the preservation of contractile myofibers but also on the coordinated organization of its extracellular matrix, vascular supply, and neural circuitry. The disruption of any of these interconnected components compromises the homeostatic and regenerative capacity of muscle, ultimately impairing functional recovery following VML.

### 3.2. Myogenic Proliferation, Differentiation, and Maturation

**Figure 3:**
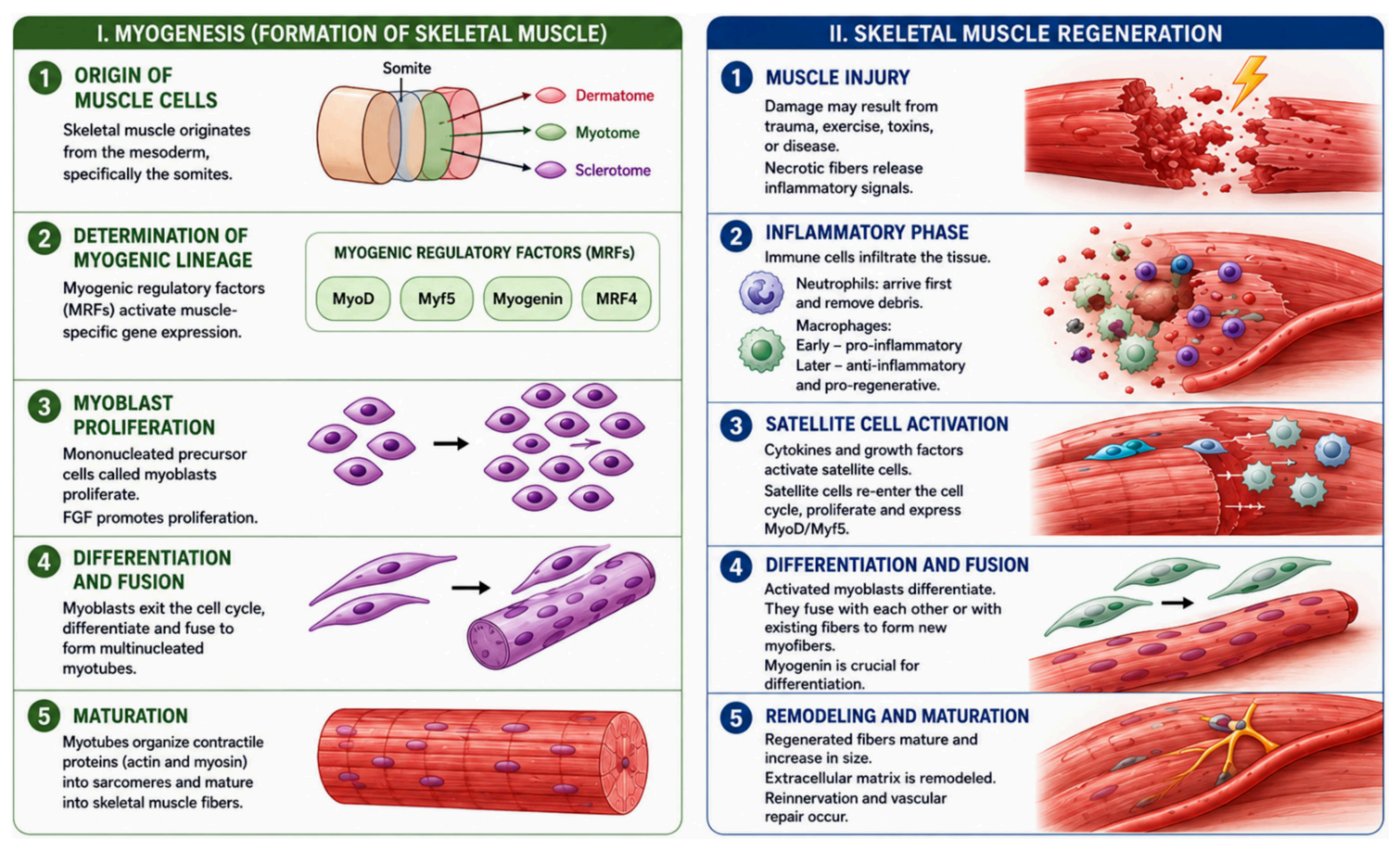
Key concepts of skeletal muscle myogenesis and regeneration. This figure was generated using the AI-assisted design tool. The initial layouts were subsequently refined, labeled, and verified by the authors to ensure scientific accuracy.

Skeletal muscle originates from mesodermal pluripotent stem cells (PSCs), which undergo a complex developmental process, including the commitment of proliferating somitic cells to the myogenic lineage, followed by the differentiation of committed myoblasts into myocytes and myotubes. The skeletal muscle development is controlled by four myogenic regulatory factors (MRFs): MyoD, Myf-5, myogenin, and MRF4, which regulate the coordinated activation of multiple muscle-specific genes during myogenesis (Figure 3) [15]. For example, MyoD and Myf-5 control the commitment of proliferating somitic cells to the myogenic lineage, whereas myogenin and MRF4 direct the differentiation of committed myoblasts into myocytes and myotubes [15].

The central role in muscle development belongs to myogenic differentiation 1 (MyoD), often called the “master switch” because it can activate the whole myogenic transcription program. The interaction between MyoD and E12 is recognized as crucial for this regulation because a forced MyoD-E12 heterodimer robustly restores differentiation in rhabdomyosarcoma cells [16]. Modified interactions of MyoD and E12 were utilized to selectively activate muscle differentiation programs and specifically influence aspects of cell biochemistry including cytoskeleton, excitability, and metabolic profiles [17,18]. This synthetic biology approach could lead to customization of pre-developed myofiber properties for specific needs in reparative medicine.

At the tissue level, skeletal muscle regeneration also depends on coordinated interactions among macrophages, endothelial cells, myogenic progenitor cells, the extracellular matrix (ECM), and nervous tissue. Restorative macrophages stimulate myogenesis and angiogenesis [14,19] by secreting pro-inflammatory cytokines and promoting coupling [14,19] between these processes, while the ECM provides a scaffold for muscle-cell adhesion and migration and nervous tissue supplies signals that support muscle growth and differentiation [20]. Together, these molecular and cellular mechanisms create the regenerative microenvironment that underlies skeletal muscle repair (Figure 3) and forms the biological basis for current volumetric muscle loss (VML) therapeutic strategies.

### 3.3. Current Clinical Approaches and Limitations

The most prevalent surgical intervention for large muscle defects is an autologous muscle flap transfer, in which a portion of a donor muscle, commonly the latissimus dorsi, rectus abdominis, gluteus maximus, or gracilis, is harvested and transposed to fill the defect [21]. While flap transfer restores soft-tissue coverage by occupying the defect site, the transplanted muscle must undergo reinnervation in order to recover active contractile function, a process that is incomplete in adults, particularly when the defect site lacks viable nerve stumps. Additionally, flap transfers impose a secondary injury at the donor site, which can itself experience a loss of function [22]. As a result, the functional outcomes following flap transfers are highly variable, with the literature reporting peak force recovery rates of 50–70%, at best, and lower reinnervation rates [23].

A superior surgical intervention, requiring considerable precision and expertise by medical specialists, is a free functional muscle transfer (FFMT), in which a portion of donor muscle is harvested along with its neurovascular pedicle and transplanted to the injury site, where it is anastomosed and coapted to local vessels and nerves, respectively [24]. In contrast to non-innervated flap transfers that primarily provide structural coverage and support, FFMT aims to re-establish muscle function by promoting axonal reinnervation and vascular ingrowth. However, as with flap transfers, the functional outcomes of FFMTs are incomplete and variable, often attributable to donor-site morbidity, prolonged denervation, suboptimal revascularization, limited integration with the ECM, and impaired reformation of the neuromuscular junction (NMJ), all of which lead to muscle atrophy [21,25,26]. While FFMT can provide partial restoration of limb contour and muscle function, peak strength recovery rates consistently fall short of native muscle performance, providing a strong rationale for the development of tissue engineering and regenerative strategies for VML.

The utilization of acellular scaffolds derived from xenogeneic or allogeneic decellularized ECM has attracted considerable attention as an alternative to autologous tissue transfer. Of these grafts, small intestinal submucosa (SIS) and urinary bladder matrix (UBM) have been the most extensively studied and are also commercially available [27,28,29]. In 2014, Sicari et al. [30] conducted a landmark small clinical series, in which five patients with musculoskeletal injuries with at least a 25% functional deficit to classify as VML received implantation of multi-laminate SIS or UBM scaffolds. On examination, electromyographic strength testing demonstrated that patients with the implants demonstrated significant improvements over the baseline, with two of five patients showing new skeletal muscle fiber formation at the implant site. However, the absolute functional gains were modest, averaging approximately 25% [30]. Additionally, the investigation was non-randomized, while also lacking a matched control cohort, thus limiting the strength of its conclusions [30]. In 2016, a multi-site case series by Dziki et al. reinforced these results by demonstrating that acellular scaffolds promote constructure remodeling of the muscle injury site, characterized by site-appropriate tissue deposition; however, they also emphasized that the magnitude in strength recovery correlates with the extent of physical rehabilitation, thereby suggesting that acellular scaffolds alone are insufficient [31].

The underlying regenerative mechanism of acellular scaffolds can be attributed to their ability to elicit an intense macrophage response, specifically a polarization towards an anti-inflammatory. Valentin et al. [32] demonstrated that acellular scaffold biomaterials, including those derived from SIS, elicited a markedly different immune response compared to other biomaterials or scaffold designs, and unexpectedly, autologous tissue grafts as well. An immunohistochemical analysis found that non-crosslinked SIS scaffolds promoted a predominantly M2 macrophage response, whereas crosslinked SIS scaffolds containing cellular components promoted a predominantly M1 macrophage response; surprisingly, autologous tissue was found to elicit a response that showed no preference for M1 or M2 [32]. Therefore, implantation of acellular scaffolds may actively promote myoblast differentiation and fusion, resulting in greater functional muscle gain than conventional autologous tissue transfers.

Physical and occupational therapies serve as indispensable adjunct treatments for the long-term management of VML, operating on the premise that consistent neuromuscular activation drives hypertrophy of muscle fibers, and subsequently, a compensatory reorganization of skeletal muscle as well as motor cortex representation. A collection of systematic reviews of rehabilitation protocols for VML have confirmed that structured exercise programs improve muscle function and force transmission when compared to controls; however, they largely fail to adequately restore mass and volume deficits [33]. This is the expected outcome as the biological mechanism of exercise satellite-cell activation through mechanical strain is itself compromised in VML.

The evaluation of pharmacological adjuncts, including myostatin, transforming growth factor-β (TGF-β), and tumor necrosis factor-α (TNF-α) inhibitors as well as insulin-like growth factor-1 (IFG-1), fibroblast growth factor (FGF), and vascular endothelial growth factor (VEGF) analogs, has been investigated in preclinical in vitro models to augment hypertrophy of existing muscle following VML, with mixed results [34,35,36,37]. A recent report by Clark et al. demonstrated that IGF-1 delivery in a muscle filler increased muscle mass; however, this structural increase did not translate to improvements in muscle fiber hypertrophy, torque production, or neuromuscular function [38]. The administration of platelet-rich plasma (PRP) has also been explored as an autologous growth factor concentrate that accelerates healing at sites of VML [39]. The mechanistic rationale for the delivery of growth factors (PDGF, VEGF, EGF, TGF-β) in supraphysiologic concentrations to stimulate cell proliferation, angiogenesis, and matrix remodeling is appealing; however, there is a lack of clinical evidence for the efficacy of PRP. Additionally, widespread variation in preparation protocols for PRP has prevented efforts for comparative analysis.

**Figure 4:**
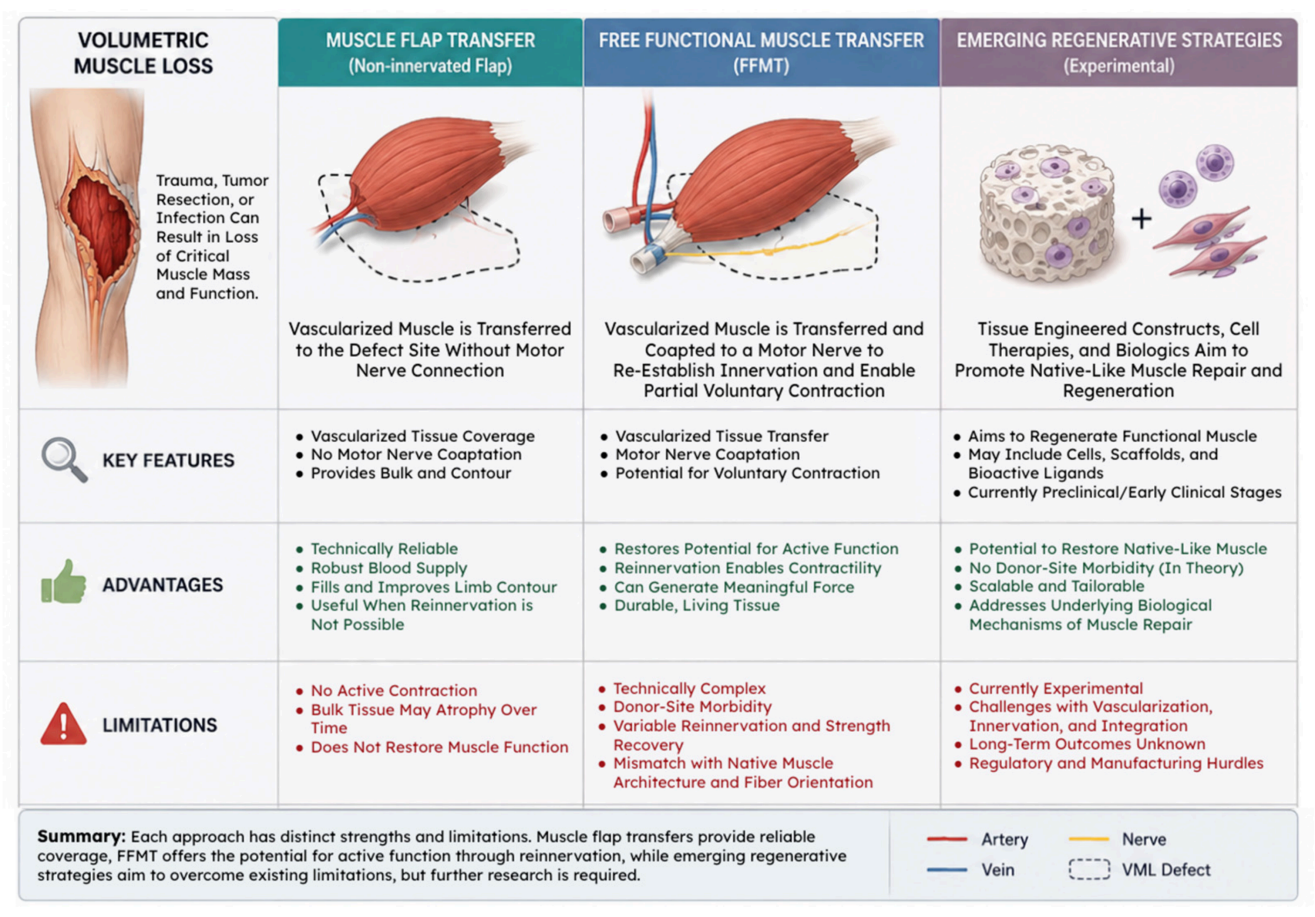
Comparison of current clinical approaches and emerging regenerative strategies for VML. “This figure was generated using the AI-assisted design tool FigureLabs. The initial layouts were subsequently refined, labeled, and verified by the authors to ensure scientific accuracy”.

Overall, current standards of care (i.e., muscle flap transfers, FFMTs, acellular scaffolds, physical rehabilitation, and pharmacological adjuncts, (Figure 4)) address one facet of the multifactorial problem of muscle regeneration for VML, leaving other critical factors unresolved.

Autologous muscle transfers restore muscle volume, but provide variable functional recovery, while introducing donor-site morbidity. Acellular scaffolds support constructive matrix remodeling but fail to regenerate adequate contractile muscle throughout the defect. Physical rehabilitation optimizes existing muscle function but fails to reestablish muscle mass and volume. Collectively, these approaches individually or combinatorially fail to achieve the benchmark of restoring muscle function to at least 80% of pre-injury baseline levels, which is widely accepted as the threshold for meaningful functional recovery [40]. Thus, these therapeutic gaps underscore the importance of developing multimodal regenerative approaches, which are discussed in the following sections.

## 4. Design Principles and Emerging Strategies in Scaffold-Based Muscle Regeneration

Scaffold-based engineering remains central to skeletal muscle regeneration; however, conventional scaffolds often fail due to insufficient vascularization, limited innervation, and inadequate mechanical reinforcement during early healing. Many prior reviews describe scaffold categories but seldom distinguish which scaffold design principles most directly address biological bottlenecks in VML. Overall, it is critical to initiate a framework linking scaffold architecture to specific regenerative outcomes. For example, aligned microchannels and nanofiber scaffolds offer topographical guidance cues that facilitate myoblast alignment, elongation, and fusion into parallel myotubes, effectively replicating the anisotropic architecture of native skeletal muscle. Several studies indicate that having aligned fibers with diameters between 300 and 1000 nm, as well as highly oriented microchannels, substantially improves myotube alignment, elevates the fusion index, and enhances the expression of myogenic markers, including MyoD, myogenin, and myosin heavy chain (MHC), compared with randomly oriented scaffolds [41,42,43,44,45]. Viscoelastic hydrogels help satellite cells maintain their quiescence, mainly due to their stress-relaxation properties. Studies show that fast-relaxing hydrogels preserve stem cell markers and quiescence better than elastic or slow-relaxing gels, which promote differentiation. Soft, fast-relaxing collagen hydrogels especially support stemness, while slower-relaxing gels favor myoblast proliferation. Hydrogels also improve the efficiency of satellite-cell transplantation. However, most research focuses on cell culture and transplantation within hydrogels, not infiltration into existing ones. Overall, viscoelasticity is crucial, but the best properties depend on the cell type and application [46,47,48,49]. Porosity and pore interconnectivity are fundamental design parameters that regulate nutrient diffusion, oxygen transfer, waste removal, and host–cell infiltration within engineered skeletal muscle constructs. Highly interconnected pore networks facilitate macrophage migration, endothelial cell invasion, and myogenic progenitor cell infiltration, consequently promoting angiogenesis and tissue remodeling. Experimental studies have further demonstrated that increasing pore size enhances myoblast infiltration and alignment, while pores exceeding approximately 200 μm support the formation of larger blood vessels and their deeper penetration, thereby improving vascular integration within regenerating tissues. Although the optimal pore architecture depends on the biomaterial and fabrication strategy, this evidence jointly indicates that highly porous, interconnected scaffolds provide a favorable microenvironment for skeletal muscle regeneration by balancing efficient mass transport with cell organization and vascularization [50,51,52,53]. Conductive scaffolds that enable the delivery of localized electrical and mechanical cues that mimic the electrophysiological environment of native skeletal muscle can enhance myogenic differentiation and tissue maturation. Studies show that materials such as polypyrrole, polyaniline, and carbon-based nanomaterials promote myoblast alignment, fusion, and maturation in conductive scaffolds compared with non-conductive controls [54,55]. For example, aligned conductive scaffolds increased myotube formation by approximately 40–80%, while conductive electrospun fibers produced a 3.6-fold increase in sarcomeric myosin-positive cells and an approximately three-fold increase in myosin heavy chain (MHC) expression [56,57]. In addition, conductive scaffolds have also been shown to upregulate key myogenic regulatory factors, including Myf5, MyoD, and myogenin, indicating enhanced myogenic commitment. Furthermore, when combined with electrical stimulation, these platforms exhibited dose-dependent improvements in muscle maturation, increasing myotube area by 1.4–2.4-fold and myotube length by 1.3–2.0-fold [58]. This finding reflects that conductive and mechano-responsive scaffolds not only provide structural support but also actively regulate myogenic differentiation and functional maturation by recreating the bioelectrical microenvironment of native skeletal muscle.

**Figure 5:**
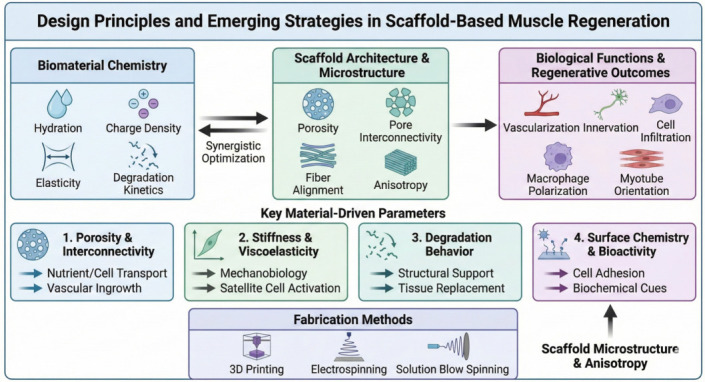
Design paradigm linking scaffold properties to regenerative muscle function. “This Figure was generated using the AI-assisted design tool FigureLabs. The initial layouts were subsequently refined, labeled, and verified by the authors to ensure scientific accuracy”.

Overall, designing scaffolds for skeletal muscle regeneration should focus on more than just choosing the right material or shape (Figure 5). It is important to align the chemistry, structure, and biological functions of the biomaterial. This approach is key to solving the ongoing challenges in VML repair. Factors such as alignment, porosity, softness, degradation rate, and surface features all influence cell attachment, cell behavior, immune response, blood vessel growth, and tissue repair. The most successful scaffolds balance these features to closely mimic real muscle. When designing a scaffold, some basics are especially important. Pore size and connectivity help with nutrient flow and blood vessel growth. The stiffness or softness of the scaffold influences how muscle stem cells activate and become muscle cells. The scaffold needs to be stable at first but gradually break down as new muscle develops. Its surface should support cell attachment and send helpful signals. Methods such as fiber spinning or 3D printing shape these features and influence how effectively the scaffold aids muscle healing.

### 4.1. Biomaterials: The Bricks to Build Regenerative Muscle Tissue

Biomaterials serve as the foundational building blocks, or “bricks”, in the architecture of skeletal muscle regeneration. Beyond their role as mere structural support, they actively guide cellular behavior, facilitate integration with host tissue, and influence the success of scaffold-based therapies. Whether natural, synthetic, or conductive, biomaterials are chosen and engineered based on critical properties such as mechanical stiffness, elasticity, degradation rate, porosity, cell adhesion, protein absorption and biocompatibility. Like bricks in construction, each material component contributes to the scaffold’s strength, flexibility, and function, forming the base upon which cellular regeneration can be built (Table 1).

Polymeric biomaterials, natural or synthetic, were considered as an alternative solution for tissue transplantation and numerous polymers were evaluated for the purpose of regenerating musculoskeletal tissues. The aim of this field is to design and engineer the muscle tissues as a graft for the damages or lost muscle. Therefore, the selection of the polymers requires a deep understanding of the tissues’ features such as mechanical properties (stiffness and elasticity) and physical features (morphology and hydrophilicity). Once these features are identified, the engineering of the scaffolds for the efficient transformation of stem cells towards the desired tissue would gain more effective and successful results. The scaffolds must be capable of inducing differentiation of the seeded stem cells, a process known as myogenic differentiation, to form myofibers alongside blood vessels and neuromuscular junctions.

Among natural polymers, collagen is a fundamental component of the extracellular matrix and exists in multiple forms that provide a range of elastic properties. It plays a crucial role in facilitating cell adhesion to substrates, promoting cellular differentiation, and aiding in the morphogenesis of tissues. Additionally, once cell–collagen interactions are established, it ensures efficient mechanical signal transmission. However, collagen’s performance heavily relies on the source of extraction, and the gelation pH can vary significantly among different collagen types [59]. Gelatin, derived from collagen hydrolysis, contains the arginylglycylaspartic acid (RGD) sequence, which promotes cell adhesion [59]. As an FDA-approved protein, it has diverse applications and can form physical gels at 35 °C, with possibilities for modification using cellulosic biomaterials to enhance gelation temperatures. Its numerous modifiable functional groups on side chains have led to the successful creation of gelatin methacrylate (GelMa). Nonetheless, gelatin has weak mechanical strength, degrades quickly, has low water solubility at room temperature, and is sensitive to high temperatures [60].

Hyaluronic acid is another important natural polymer that facilitates cell adhesion, growth, migration, and differentiation. Hyaluronic acid is a disaccharide polymer made of D-glucuronic acid and N-acetyl-D-glucosamine. It is a major component of the ECM in connective tissues and is highly hydrophilic, largely due to the presence of hydroxyl groups and negatively charged carboxylic acid groups. The balance of these acids influences the polymer’s rigidity and biocompatibility, although it exhibits a high swelling ratio and low mechanical strength [61].

Alginate, a copolymeric acid formed from repeating units of α-L-glucuronic acid and β-D-mannuronic acid, varies in molar ratios depending on the extraction source. The anionic nature of alginate allows for reversible physical gel formation in the presence of multivalent cations like Ca^2+^ [62]. However, its dimensional stability is weak, and it shows low heat resistance above 60 °C, leading to rapid degradation during processing. Furthermore, alginate precipitation can occur at low pH levels, limiting the control over the mechanical and morphological properties of scaffolds [63].

Chitosan, the deacetylated form of chitin, is known for its non-inflammatory and bioresorbable products resulting from hydrolysis. It possesses antibacterial properties due to its positively charged nature, which also facilitates the controlled release of growth factors that are anionic [64]. Unfortunately, chitosan suffers from poor mechanical strength and thermal stability, which can limit its applicability in various scaffolding scenarios [65].

In addition to structural and biochemical cues, electrical stimulation plays an essential role in regulating the alignment, proliferation, and differentiation of electrically responsive cells such as myoblasts and cardiomyocytes. These cells capable of orientation in the presence of electrical stimuli and field mainly include cardiomyocytes and myoblasts. Apart from cell orientation, the continuous electrical pulses are also able to facilitate cell migration and proliferation. The electrical field/continuous pulse and the specially designed porous morphology of the scaffold can encourage the myogenic progenitors to form the main components of the skeletal muscle. Despite their potential in conducting electrical current or pulse, they are commonly used in the form of dispersed phase in synthetic and non-conducting polymer matrices. When selecting the matrix polymer to design the scaffold, the rate of the hydrolytic degradation, mechanical strength and elasticity are the primary features of the materials that are considered.

Conducting nanomaterials, mainly carbon-based nanoparticles such as CNTs, were investigated in the last decade for their potential as a conducting filler in bioactive scaffolds [66]. However, their application was limited due to the concerns over their toxicity and hardships of controlling their distribution in polar polymer matrices such as hydrogels. Conductive polymer, however, gained interest in replacing the conducting nanoparticles as their chemistry and synthesis methods progressed. The electrical conductivity of these polymers resembles that of metal-based semiconductors.

The main conductive biocompatible polymers widely used for regeneration of electrically sensitive tissues are polypyrrole (PPY), polyaniline (PANI), and polythiophene. The use of these conductive polymers in the substrate allows for electrical stimulation of the cultured cells using continuous electrical pulses. Hence, the scaffold acts as a bioactive substrate stimulating the orientation of the myoblasts to further develop into myocyte and myofiber. The conductive polymers are highly biocompatible and support cell adhesion and proliferation, and even in the absence of electrical current and pulse, they can promote differentiation of C2C12 cells [67].

**Table 1:** Comparison and review of biomaterials used in scaffold fabrication for VML.

Biomaterial	Key Advantages	Primary Limitations	Typical Modifications	Representative Applications in Skeletal Muscle Engineering	References
Fibrin	➤ Highly Biomimetic ➤ Supports Myoblast Adhesion and Satellite Cell Activation ➤ Enzymatically Degradable ➤ Autologous Source ➤ Promotes Angiogenesis and Hemostasis	➤ Low Mechanical Strength ➤ Rapid Degradation ➤ Prone to Scaffold Contraction ➤ Limited Long-Term Structural Stability and Integration with Native Tissue	➤ Genipin or Thrombin Crosslinking ➤ Composite Hydrogels with Collagen, PCL, or GelMA ➤ Fiber Reinforcement	➤ Injectable Hydrogels ➤ Cell Delivery Vehicles ➤ Aligned Muscle Constructs	[68,69,70]
Collagen/Gelatin	➤ Major ECM Component ➤ Intrinsic Cell-Adhesive Ligands ➤ Excellent Biocompatibility ➤ Autologous Source ➤ Gelatin Offers Tunable Chemistry	➤ Batch-to-Batch Variability ➤ Relatively Weak Mechanical Properties ➤ Rapid Enzymatic Degradation ➤ Gelatin Properties Highly Dependent on Source and pH	➤ Fiber Alignment ➤ Composite Hydrogels with GelMA, GelNB, GelSH ➤ Chemical or Photo-Crosslinking	➤ Aligned Scaffolds ➤ 3D Bioprinting ➤ Hydrogel Bioinks	[60,71,72,73,74,75]
Alginate	➤ Tunable Stiffness ➤ Good Printability ➤ Suitable for Cell Encapsulation and Controlled Factor Delivery	➤ Bioinert; Lacks Intrinsic Cell-Adhesion Ligands ➤ Slow and Poorly Controlled Degradation ➤ Limited Myogenic Support Without Modification	➤ RGD Functionalization ➤ Blending with Collagen, Gelatin, or Fibrin ➤ Composite Hydrogels ➤ Growth Factor Incorporation	➤ Injectable Hydrogels ➤ Bioprinted Constructs ➤ Stem Cell and Drug Delivery	[76,77,78,79]
Chitosan	➤ Biocompatible and Biodegradable ➤ Antimicrobial and Anti-Inflammatory ➤ Structurally Similarly to Glycoaminoglycans (GAGs) ➤ Supports Wound Healing	➤ Poor Mechanical Strength ➤ Limited Solubility at Physiological pH ➤ Relatively Weak Cell Adhesion ➤ Variable Degradation	➤ Blending with Collagen, Gelatin, Alginate, or PCL ➤ Chemical or Photo-Crosslinking ➤ Nanoparticle Incorporation	➤ Composite Scaffolds ➤ Wound Healing Matrices ➤ Drug Delivery	[65,80]
Hyaluronic Acid (HA)	➤ Native ECM GAG ➤ Regulates Cell Migration and Proliferation ➤ Highly Hydrated ➤ Promotes Angiogenesis and Tissue Remodeling	➤ Very Low Mechanical Strength ➤ Rapid Degradation ➤ Limited Structural Support and Integration	➤ Methacrylation (HAMA) ➤ Chemical Crosslinking ➤ Composite Scaffolds ➤ Growth Factor Incorporation	➤ Injectable Hydrogels ➤ Bioinks ➤ Stem Cell Delivery ➤ Regenerative Matrices	[81,82]
Decellularized Extracellular Matrix (dECM)	➤ Preserves Native ECM Composition, Architecture, and Bioactive Signaling Molecules ➤ Tissue-Specific Biochemical Cues ➤ Highly Biomimetic ➤ Promotes Myogenic Differentiation	➤ Donor Tissue Variability ➤ Incomplete Decellularization Risk ➤ Limited Mechanical Strength ➤ Difficult Standardization and Scale-Up	➤ Tissue-Specific Decellularization Protocols ➤ Composite Scaffolds ➤ Methacrylated dECM Bioinks ➤ Reinforcement with Synthetic Polymers	➤ Tissue-Specific Bioinks ➤ Injectable Hydrogels ➤ Engineered Skeletal Muscle Constructs	[83,84]
Conductive Nanoparticles	➤ Improves Electrical Conductivity ➤ Enhances Electrical Signal Propagation ➤ Promotes Myogenic Differentiation and Maturation Under Electrical Stimulation	➤ Potential Cytotoxicity ➤ Aggregation Tendencies ➤ Non-Uniform Dispersion ➤ Uncertain Long-Term Safety In Vivo	➤ Incorporation Into Polymer Matrices ➤ Surface Functionalization ➤ Hybrid Conductive Composites	➤ Electroactive Scaffolds ➤ Biosensing ➤ Electrically Stimulated Muscle Tissue Engineering Constructs	[85,86]
Conductive Polymers	➤ Intrinsic Electrical Conductivity ➤ Enhances Myotube Maturation and Contractile Function ➤ Tunable Electrical Properties	➤ Limited Biodegradability ➤ Potential Biocompatibility Concerns ➤ Reduced Processability When Used Alone	➤ Blending with Biodegradable Polymers ➤ Surface Coating ➤ Copolymerization	➤Electroconductive Scaffolds ➤Electrically Responsive Hydrogels ➤ Neuromuscular Tissue Engineering Constructs	[67,87]

### 4.2. Three-Dimensional Bioprinting

The emergence of three-dimensional (3D) bioprinting has enabled precise spatial deposition of biomaterials, cells, growth factors, and even vascular channels with resolutions approaching the scale of individual muscle fascicles [88]. Unlike conventional molding or electrospinning approaches, bioprinting can encode complex geometrical information, such as the macro-scale shape of the defect, meso-scale organization of fascicles, and micro-scale network of vessels, into a single fabrication workflow.

**Figure 6:**
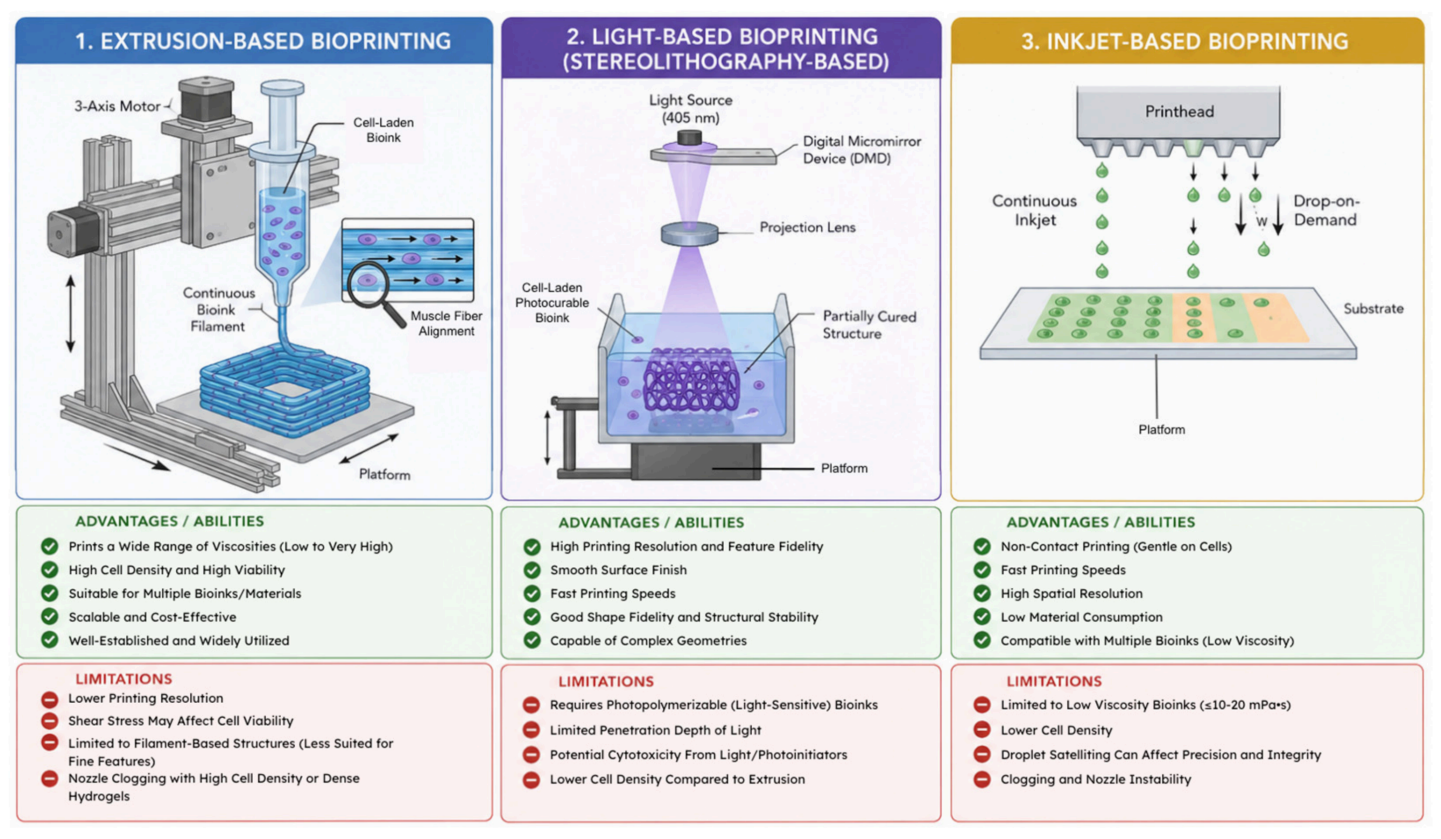
Comparison of the three major bioprinting technologies for skeletal muscle tissue engineering. “This figure was generated using the AI-assisted design tool FigureLabs. The initial layouts were subsequently refined, labeled, and verified by the authors to ensure scientific accuracy”.

The current bioprinting landscape consists of three principal fabrication techniques: extrusion, light-assisted, and inkjet bioprinting (Figure 6). Extrusion-based bioprinting deposits bioink, a printable formulation of hydrogel, cells, or polymers, through a nozzle under mechanical or pneumatic pressure [89]. The configuration for these printers typically consists of a nozzle with syringe attachment, a motor with three axis motion, and a platform on which the scaffold is constructed. For muscle tissue engineering, bioinks must balance printability factors (i.e., sufficient viscosity to maintain filament structure post-deposition) with cell viability (i.e., minimal shear stress during extrusion to prevent mechanically induced cell death). Costantini et al. demonstrated that the coaxial extrusion of a hydrogel fabricated from PEG-Fibronogen laden with myoblasts produced myofiber analogs that matured into aligned, contractile myotubes when implanted in immunocompromised mice, which represented a significant benchmark for bioprinted muscle constructs [88]. Additionally, Kim et al. leveraged extrusion-based bioprinting to produce a gelatin hydrogel containing human muscle progenitor cells and PCL, which produced significant functional recovery in tibialis anterior muscle defects in a rodent model of 82% [90]. GelMA, a gelatin composite that is photo-crosslinkable by ultraviolet (UV) light, has become widely adopted as an optimal bioink base because its stiffness can be tuned between 1 and 40 kPa by varying the concentration and crosslink exposure time [91,92]. In a recent investigation, Hwangbo et al. fabricated a composite scaffold containing GelMA laden with myoblasts and adipose-derived stem cells, which produced significantly greater muscle strength recovery when implanted in a mouse model [92].

Light-assisted bioprinting, particularly stereolithography-based bioprinting (SLA), crosslinks entire layers at once through photopolymerization, enabling faster production of complex geometries. Grigoryan et al. utilized stereolithography to fabricate intravascular helices within a hydrogel composed of PEGDA, demonstrating that sacrificial carbohydrate templates could encode branching vascular networks [93]. Although this investigation was not applied to muscle, follow-up works have integrated light-assisted bioprinting for fabricating vascular templates within myogenic hydrogels [94,95]. While light-assisted bioprinting allows for greater efficiency and resolution, its applications in muscle tissue engineering are limited by photoinitiator cytotoxicity, light-associated cytotoxicity, and limited scale, particularly for the centimeter-scale constructs relevant to VML.

Inkjet bioprinting ejects picoliter droplets through thermal or piezoelectric actuators, offering considerably greater resolution than extrusion-based bioprinting at the cost of strict viscosity limits. Inkjet bioprinting can be subdivided into continuous and drop-on-demand (DoD) inkjet; continuous inkjet ejects bioink in a continuous, high-pressure stream that breaks into droplets, while drop-on-demand inkjet ejects ink in single droplets, only when required [96]. While inkjet bioprinting eliminates nozzle contact, thereby minimizing the shear stress applied during the printing process, its applications in muscle tissue engineering are limited by droplet discontinuity, poor mechanical properties, and inconsistency with native tissue [97]. Additionally, strict viscosity limits preclude the majority of cell-laden hydrogels and droplet discontinuity may induce potential losses in cell viability [98].

The major challenge for bioprinting of functional muscle, or constructs that promote regeneration of functional muscle, is the discrepancy between printed construct architecture and native tissue organization. As previously discussed, native skeletal muscle organizes myofibers into fascicles, fascicles into whole muscles with complex pennation angles (the internal angle at which a fascicle aligns with a muscle’s line of action), and muscles into groups with myotendinous insertions and neuromuscular junctions. Current bioprinting techniques are unable to reliably reproduce the pennation angles or neuromuscular junctions at scale, although multi-material bioprinters have begun to address these challenges. The integration of machine learning for bioink formulation optimization is emerging as technology continues to systematically bridge the gap.

### 4.3. Nanofiber Technology and Background

Nanofibers have become one of the most promising scaffold architectures for skeletal muscle tissue engineering because they closely mimic the fibrous organization of the native extracellular matrix (ECM). Rather than serving solely as structural materials, nanofibers provide topographical and mechanical cues that regulate myoblast attachment, alignment, migration, proliferation, and differentiation, rendering them especially attractive for the regeneration of volumetric muscle loss (VML).

Nanofibers are characterized by high surface-to-volume ratio and microporous structno needure. Nanofibers are solid fibers that have two exterior dimensions of nanometers with a diameter less than 100 nm and a fiber length greater than the diameter. A nanofiber is composed of tens to hundreds of nanometer-sized ultra-fine fibers possessing specific physicochemical characteristics [99]. The diameter of nanofibers can vary and be modified depending on the polymer, spinning technique, and spinning parameters used. The quality of the nanofibers depends on the diameter, shape, and surface texture of fiber. In addition to specific surface area, fiber shape (hollow or core–shell) and diameter provide flexibility in tailoring nanofiber attributes. Fabrication and utilization of nanofibers have become a growing topic in research. Nanofibers have different exceptional attributes beyond high surface area like good permeability, and enhanced physical, mechanical, and biocompatible properties. Natural and synthetic biopolymers can both be used to manufacture nanofibers. Nanofibers can be produced from but are not limited to natural polymers like collagen, silk fibroin, gelatin, keratin, chitosan, celluloses, and synthetic polymers like polylactic acid (PLA), poly (lactic-co-glycolic acid) (PLG/PLGA) and polycaprolactone (PCL), polyurethane (PU) [100]. Natural polymers have inherent increased biocompatibility and biological activity compared to synthetic polymers. Although this is a great plus for natural polymers, synthetic polymers generally offer better mechanical qualities and biostability properties that can be manipulated and tailored based on the application. The combination of natural and synthetic materials to construct complexes/scaffolds is an attractive strategy to retain increased biological activity without compromising mechanical characteristics and biostability [101]. From a regenerative perspective, anisotropic architecture is the most valuable feature because its potential to enhance myoblast alignment and fusion into parallel myotubes, thereby replicating the organization of native skeletal muscle, is of great value. The most common fabrication methods of nanofibers are electrospinning and Solution-Blow or Air-jet spinning.

### 4.4. Electrospinning Nanofibers

**Figure 7:**
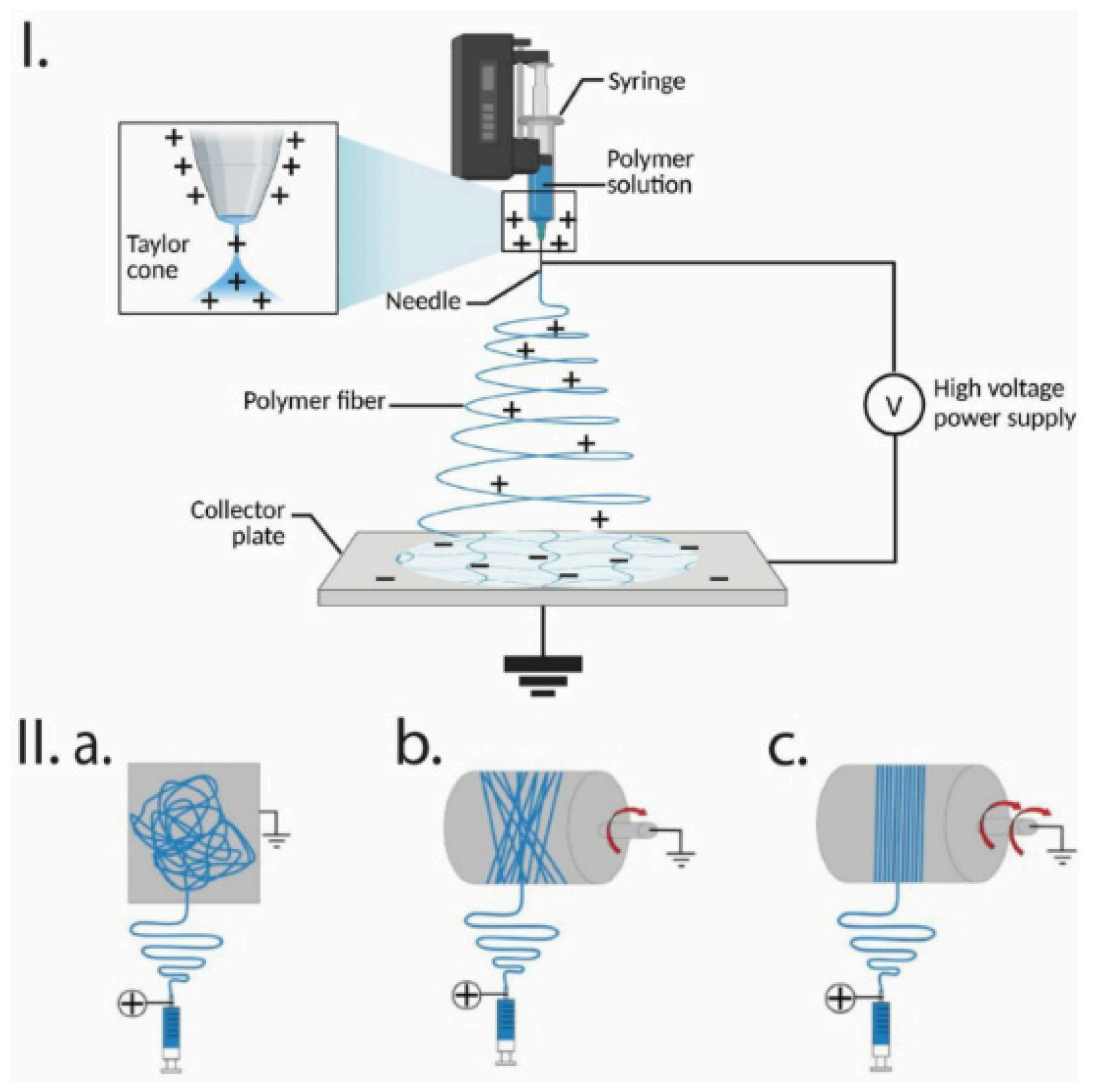
Overview of electrospinning for generating random and aligned nanofibers. (**I**) During electrospinning, a high voltage is applied to a liquid droplet formed by the polymer solution, which results in the elongation of the droplet at the tip of the needle because of the overpowering of the surface tension by the large repulsive force. A “Taylor cone” is formed at a threshold voltage, leading to liquid discharge from the needle that eventually gets deposited on the collector in the form of fibers. (**II**) Generation of randomly aligned fibers using (**a**) a flat plate collector or (**b**) a drum collector rotating at low speeds. (**c**) Highly aligned fibers are obtained when the drum collector is rotating at high speeds (Reprinted from [104]).

Electrospinning (ES) is a widely adopted electrostatically driven technique that produces nanofibers because of its efficiency and adaptability [102]. This method can control the size and morphology of nanofibers beneficial for synthesis of tissues engineering scaffolds. Almost every soluble polymer and additive can be used for electrospinning and can fabricate various shapes and sizes with a varying range of physiochemical properties. Nanofibers provide a suitable microenvironment for cell behaviors like attachment, migration, proliferation, and differentiation. They can mimic morphological characteristics of an ECM [99]. These properties of electrospinning allow easy control of distribution and release of the bio-functional moieties within the nanofibers by manipulating these characteristics. An electrospinning setup is composed of a few major components such as high voltage power supplier, a syringe pump, a spinneret or a needle with a blunt tip, and a collector [102]. The fabrication of nanofiber scaffolds is dictated by the polymer of choice and suitable solvent to prepare polymer solution along with drugs, peptides, and nanoparticles. Due to the high voltage applied to the liquid polymer (Figure 7)., the spinneret ejects the endless jet strands towards the grounded collector. The interfacial tension of the polymer droplet is controlled by an applied electric field. Then the droplet is elongated to form a cone called “Taylor cone” and dislodged from the cone to form a fiber jet [103]. Once a Taylor cone is achieved, it leads to the formation of the nonwoven web due to the evaporation of the solvent in the jet. Although the formation of a Taylor cone is fundamental to the electrospinning process, the clinical significance of this phenomenon lies in its ability to generate highly uniform, aligned nanofibers that can direct cellular organization, rather than in the physical mechanism itself. Therefore, optimization of electrospinning parameters is ultimately aimed at producing reproducible scaffolds with architectures that enhance muscle regeneration rather than simply controlling fiber formation.

## 5. Cellular and Exosome-Based Therapies, Drug Delivery Strategy

### 5.1. Stem Cell Therapies

A limitation of the common practice of using muscle from donor sites in tissue replacement therapy for volumetric muscle loss is that it is restricted to small injuries. To overcome this limitation, many researchers have fallen on natural and synthetic biocompatible biomaterials to create 2D and 3D tissue scaffolds. Hydrogels, nanoparticles, and cell seeding are used to enhance the tissue scaffolds where the biomaterials lack in facilitating the biophysical and biochemical processes involved in skeletal muscle regeneration. In most cases, such as VML, a scaffold is needed to bridge or fill in the gap between tissues. Wu et al. [105] set out to evaluate the therapeutic potential of human iPSCs seeded within fibrin hydrogels. The human iPSCs were differentiated into skeletal myogenic progenitors before being mixed into the fibrinogen solution, creating a cell-gel mixture that was applied directly into the excised region of a murine tibialis anterior muscle defect. The cell-gel mixture showed efficient differentiation into myogenic progenitors, integration of fibers between donor and host-derived cells, a neuromuscular junction NMJ formed on donor-derived muscle fibers with peripherally located nuclei indicating maturation and innervation, restoration of donor-derived pax7+ satellite cells in the treated area, and improved muscle functionality. This study manages to confirm that human iPSCs combined with fibrin hydrogel to create a 3D tissue scaffold was a viable approach to treating volumetric muscle loss in a murine model [105].

Apart from human iPSCs, stem cells can also be derived from human embryonic stem cells (hESCs). Both stem cell lineages require specific growth factors to induce proliferation and differentiation. Growth factors such as Pax7, MyoD, Pax3, and Myf5 emulate the in utero environment of growth [106]. It has been seen that these stem cells can then be transplanted into injured tissue. Murine models show transplantation induced accelerated vascularization while also inhibiting atrophy and fibrosis [107]. While there is much literature on the use of stem cells in skeletal muscle regeneration, the only FDA-approved stem cell therapy is indicated for hematopoietic stem cell transplantation. Despite this, there are current clinical trials for skeletal muscle stem cell therapies, one of which is indicated for Duchenne Muscular Dystrophy [107].

### 5.2. Role of Exosomes in Muscle Repair

Exosomes are small extracellular vesicles that mediate intercellular communication by transferring proteins, lipids, and RNAs (including miRNAs and mRNAs) to recipient cells [108,109]. In skeletal muscle, exosomes derived from mesenchymal stem cells (MSCs), adipose-derived stem cells (ADSCs), and myogenic progenitor cells (MPCs) have been shown in preclinical studies to promote myogenesis, satellite-cell activation, angiogenesis, and reduced fibrosis, including in volumetric muscle loss models [110]. Across the studies identified here, the overall pattern is favorable but clearly heterogeneous: effects vary with vesicle source and cargo, and some papers emphasize that responses still need in vivo validation or differ by tissue origin and metabolic context [111,112]. Taken together, the field is promising as a cell-free strategy for muscle repair, but it remains preclinical and not yet standardized [113]. The following subsections examine how exosomes contribute to muscle regeneration through satellite-cell activation, immune modulation, angiogenesis, and support of aged muscle.

#### 5.2.1. Exosomes and Muscle Satellite Cells

**Figure 8:**
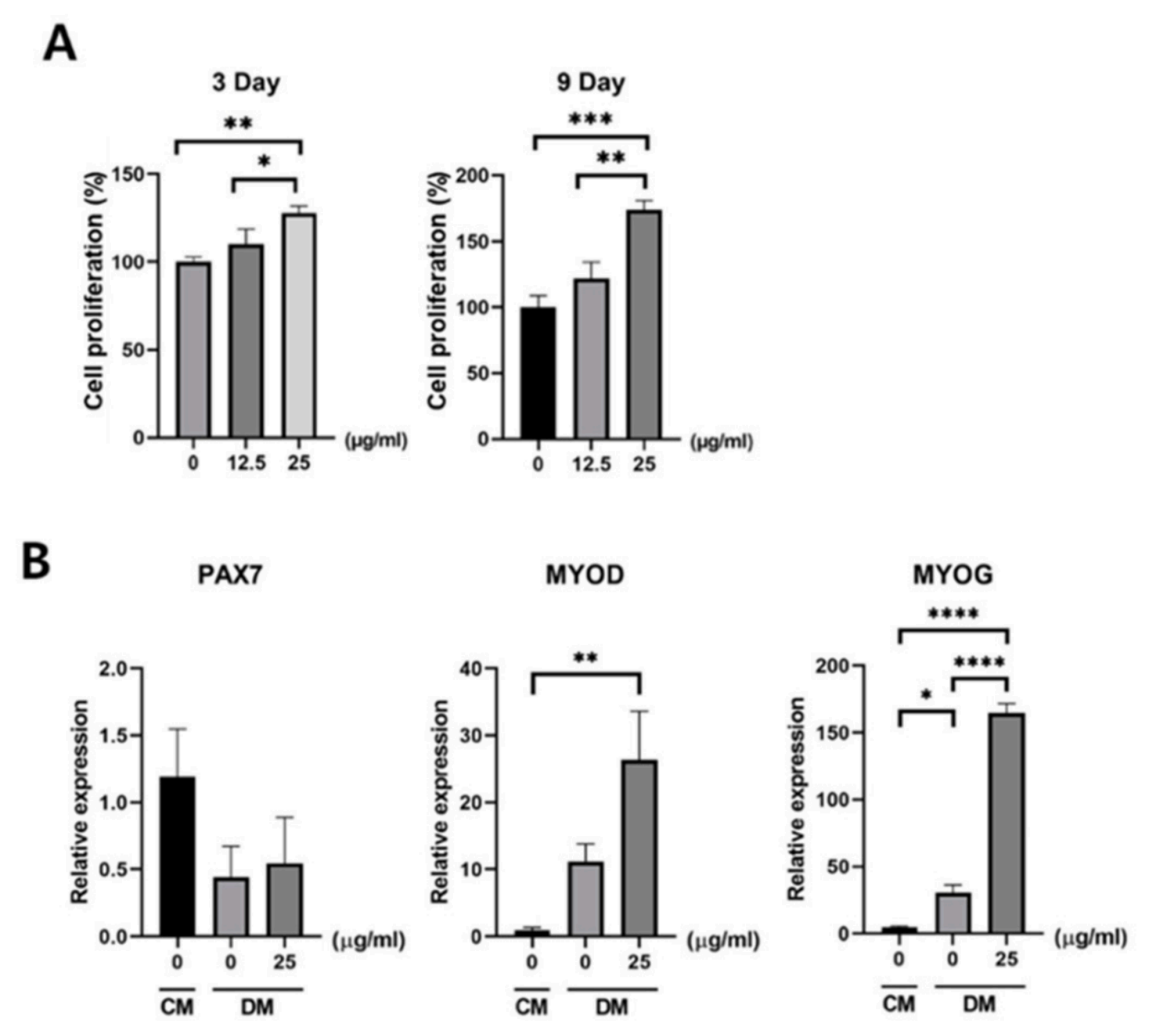
Changes in satellite-cell proliferation and differentiation markers after exosome treatment. (**A**) Exosome treatment for each satellite-cell culture (0, 12.5, and 25 μg/mL). Cell proliferation was measured using the CCK-8 solution method at 3 and 9 days after treatment. The satellite cells treated using 25 μg/mL of exosomes showed a significantly higher proliferation than that of the control both 3 and 9 days after treatment. (**B**) The qRT-PCR showed that MYOD was significantly increased by 25 μg/mL of exosomes treatment compared with those without exosome treatment in differentiation media (DM). MYOG also increased by almost 41-fold, which implies that satellite cells successfully differentiated into myocytes. The PAX7 gene decreased in DM compared with the control media. However, no statistical significance was observed; data are presented as the mean ± SD. * *p* < 0.05, ** *p* < 0.01, *** *p* < 0.001, and **** *p* < 0.0001 (*n* = 3) (Reprinted from [110]).

**Figure 9:**
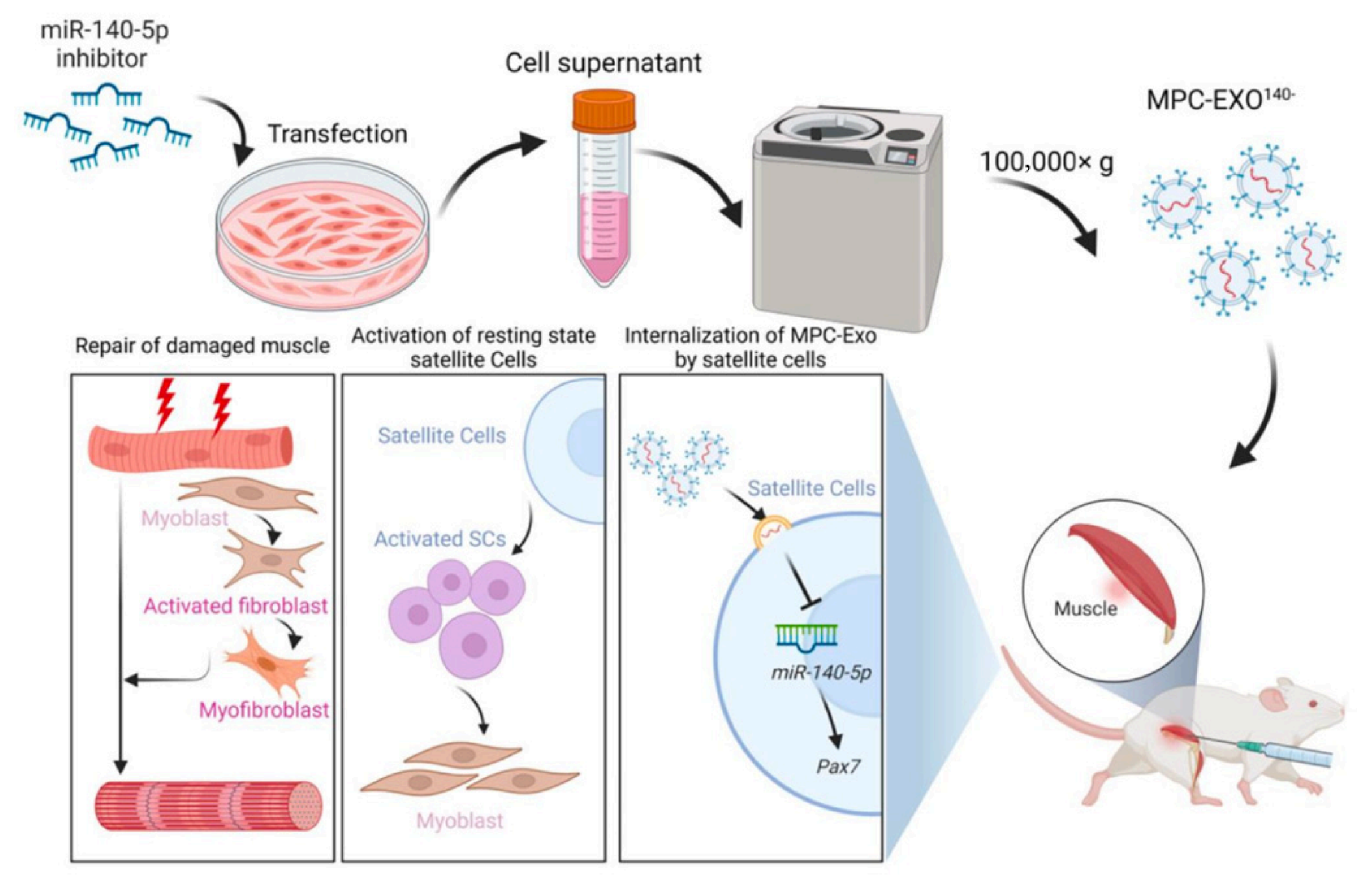
MPC−Exo140−can activate dormant muscle satellite processes, ultimately leading to the formation of new skeletal remodeling (Reprinted from [115]).

Satellite cells (SCs) play a pivotal role in the regeneration and restoration of skeletal muscle following injury. As the primary adult stem cells of skeletal muscle, SCs, remain quiescent under homeostatic conditions but rapidly activate after damage, proliferate, differentiate into myogenic progenitors, and fuse to form new myofibers while also self-renewing to maintain the stem cell pool [114]. Exosomes appear to support several of these steps, although the reported effects vary by vesicle source and molecular cargo [115,116]. MSC-derived exosomes, for example, increase satellite-cell proliferation and promote myogenic differentiation, as reflected by higher MYOD and MYOG expression and a decline in PAX7, which is consistent with activation and progression out of quiescence Figure 8 (A&B) [110]. In parallel, MPC-derived exosomes carrying a miR-140-5p inhibitor increase Pax7 expression and drive satellite cells from G1 into S phase, supporting proliferation and downstream repair as shown in Figure 9 [116,117]. C2C12 myoblast-derived exosomes have shown similar regenerative effects in injured muscle, including increased Pax7 and PCNA expression in vivo [111].

Taken together, these studies suggest that exosomes can shift satellite cells toward a more regenerative state, but the evidence is still fragmented. Different studies use different vesicle sources, purification methods, concentrations, and outcome markers, making direct comparison difficult. It is therefore not yet clear which exosomal cargo profile is optimal for promoting proliferation versus differentiation, or whether these effects generalize across injury types and species.

#### 5.2.2. Modulation of Inflammation, Immune Cell Behavior, and Fibrosis

Skeletal muscle regeneration is tightly coordinated with the immune response, where early infiltration of neutrophils and M1 macrophages clears debris, followed by a phenotypic switch to M2 macrophages that supports satellite-cell activation, myofiber repair, and tissue remodeling. Dysregulated or prolonged inflammation can impair regeneration and promote fibrosis through sustained activation of pro-inflammatory cytokines and TGF-β-mediated signaling. In this context, exosomes have emerged as critical immunomodulatory mediators that fine-tune the inflammatory milieu, promote macrophage polarization toward pro-repair phenotypes, and suppress pro-fibrotic pathways, thereby enhancing functional muscle recovery [110,118]. In a mouse skeletal muscle contusion model, bone marrow stromal cell-derived exosomes (BMSC-Exo) reduced inflammatory cytokines, decreased fibrosis, improved histologic regeneration, and enhanced biomechanical recovery. Mechanistic experiments suggested that these benefits depended in part on macrophages, because macrophage depletion abolished the therapeutic effect [119]. Other work showed that exosomal miR-223 targets IKKα and suppresses NF-κB signaling, linking vesical cargo to reduced TNF-α production and lower inflammatory activity [120,121]. Comparable anti-fibrotic effects have been reported with PRP-derived exosomes, MSC-derived exosomes, and C2C12 myoblast-derived exosomes. These preparations improved functional recovery after muscle strain or injury and reduced markers associated with fibrosis, including TGF-β, collagen I, and α-SMA [111,119,122].

The general trend is therefore toward immunomodulation and reduced scar formation, but the field still lacks consensus on how much of this benefit comes from direct effects on immune cells versus indirect effects on parenchymal muscle cells. It also remains uncertain whether the same anti-inflammatory profile can be reproduced reliably across different exosome preparations, since source material, donor state, and isolation protocol can all alter cargo composition and potency.

#### 5.2.3. Exosomes and Angiogenesis

Exosome-mediated repair: Effective regeneration also requires vascular remodeling, and exosomal cargo contributes to angiogenesis by acting on endothelial cells and pro-angiogenic signaling pathways. Several miRNAs, including miR-132, miR-146a, and miR-494, have been linked to enhance endothelial proliferation, tube formation, and capillary density through VEGF-related and PI3K/Akt signaling [115,122,123,124]. In this way, exosomes support the vascular component of repair, helping restore oxygen and nutrient delivery to injured muscle and thereby creating a more permissive environment for myogenesis [124]. This angiogenic literature strengthens the case that exosomes act as coordinators of tissue repair rather than as single-pathway agents. At the same time, much of the evidence comes from small animal studies, and the reported mechanisms may not translate directly to human muscle injury. It is also not yet clear whether angiogenesis is a primary therapeutic action of exosomes or a downstream consequence of broader changes in inflammation and regeneration.

#### 5.2.4. Exosomes and Rejuvenating Effects on Aged Skeletal Muscle

Exosomes have also been investigated as modulators of age-related decline in skeletal muscle. Systemic administration of young plasma-derived sEVs into aged animals has been shown to ameliorate age-associated declines in mitochondrial energy metabolism, in part by increasing PGC-1α expression, and supporting mitochondrial biogenesis, oxidative metabolism, ATP production, and respiratory capacity [125]. Similarly, MSC-derived exosomes have been shown to improve mitochondrial quality control in aged muscle through pathways involving Sirt1/PGC-1α, and autophagy, while also reducing apoptosis and supporting muscle mass and function [126].

These findings suggest that exosomes may influence not only acute injury repair but also the broader biology of muscle aging. However, the evidence remains largely preclinical, and the relationship to human sarcopenia or frailty is still uncertain. The studies also differ in vesicle source, dose, and route of administration, which makes it difficult to determine whether the observed effects reflect a general rejuvenating property of exosomes or a narrower effect of specific cargo in selected models.

#### 5.2.5. Exosome-Loaded Scaffolds for Skeletal Muscle Regeneration

A major challenge for free exosome therapy is that vesicles are rapidly cleared, poorly retained at injury sites, and difficult to deliver in a controlled manner. To address these limitations, exosome-loaded scaffolds have been developed using hydrogels, decellularized extracellular matrix, and synthetic polymer matrices. These biomaterial platforms can act as local reservoirs that sustain release, improve their retention, and preserve bioactivity while also providing a structural environment that supports cell infiltration, angiogenesis, and tissue remodeling [127]. In principle, this approach is attractive because it combines the signaling activity of exosomes with the mechanical and spatial advantages of a regenerative scaffold [128,129].

At the same time, this is the part of the field where translational uncertainty is most visible. Scaffold performance depends on mechanical strength, porosity, and degradation of kinetics, all of which influence release profiles and vesicle stability. The exosome source, isolation method, and loading procedure can change vesicle composition and batch-to-batch potency, making reproducibility a major concern. In vivo stability, immunogenicity, and the best route of delivery also remain unresolved. Local scaffold-based delivery may improve retention, but systemic, intramuscular, and perilesional strategies may not be equivalent in distribution, duration, or safety. For that reason, scaffold studies are important not only because they improve delivery, but because they highlight the central barriers that still limit clinical translation: standardization of exosome manufacture, consistent characterization of cargo, and proof that therapeutic activity can be preserved after formulation and delivery.

Overall, the literature supports a coherent preclinical picture in which exosomes promote muscle repair through multiple complementary mechanisms, including satellite-cell activation, immunomodulation, anti-fibrotic signaling, angiogenesis, and metabolic support. The main limitation is not the absence of positive findings, but the lack of standardization and head-to-head comparison across studies. Future work needs to define which exosome source is most effective, how isolation and storage affect potency, how long vesicles remain active in vivo, and which delivery route best balances efficacy, safety, and reproducibility.

## 6. Bio-Functional Muscle Stimulation Approaches

### 6.1. Mechanical Stimulation and Electrical Stimulation

Mechanical stimulation recreates the natural strains and stress that occur during normal daily movement while electrical stimulation is used to induce muscle contractions simulating normal activation and deactivation of motor neuron electrical synapses. The lack of either form of stimulation typically leads to muscle atrophy, whereas implementing both promotes myoblast fusion, maturation, alignment and contractility function of skeletal muscle tissue [104]. As discussed earlier, biocompatible conductive biomaterials such as polyaniline, polythiophene, and polypyrrole can be used to create tissue scaffolds that can be electromechanically stimulated. Nanoparticles such as silver, gold and graphene can be incorporated to add conductive properties to non-conductive biomaterials. Calero-Castro et al [130]. were able to produce aligned Poly(ε-caprolactone)–Gelatin Electrospun Scaffolds that performed better in guiding cell alignment, myotube formation and maturation compared to randomly oriented scaffolds. Mechanical stimulation was applied to the scaffold using a bioreactor that facilitated controlled stretching mimicking natural movement. This mechanical stimulation notably improved myotube thickness, cell viability, and the fusion index of nuclei within myotubes, correlating to more mature and differentiated muscle fibers. Electrical stimulation was also applied to the tissue scaffold at various frequencies, where lower frequencies (0.2–1.5 Hz) showed peaks in calcium transient activity which are essential for muscle contraction and functionality correlating with cell maturation. This group noted that it was beneficial to electrically stimulate the scaffold following mechanical stimulation showing a complementary effect.

### 6.2. Bioreactor for Myocyte Maturation

**Figure 10:**
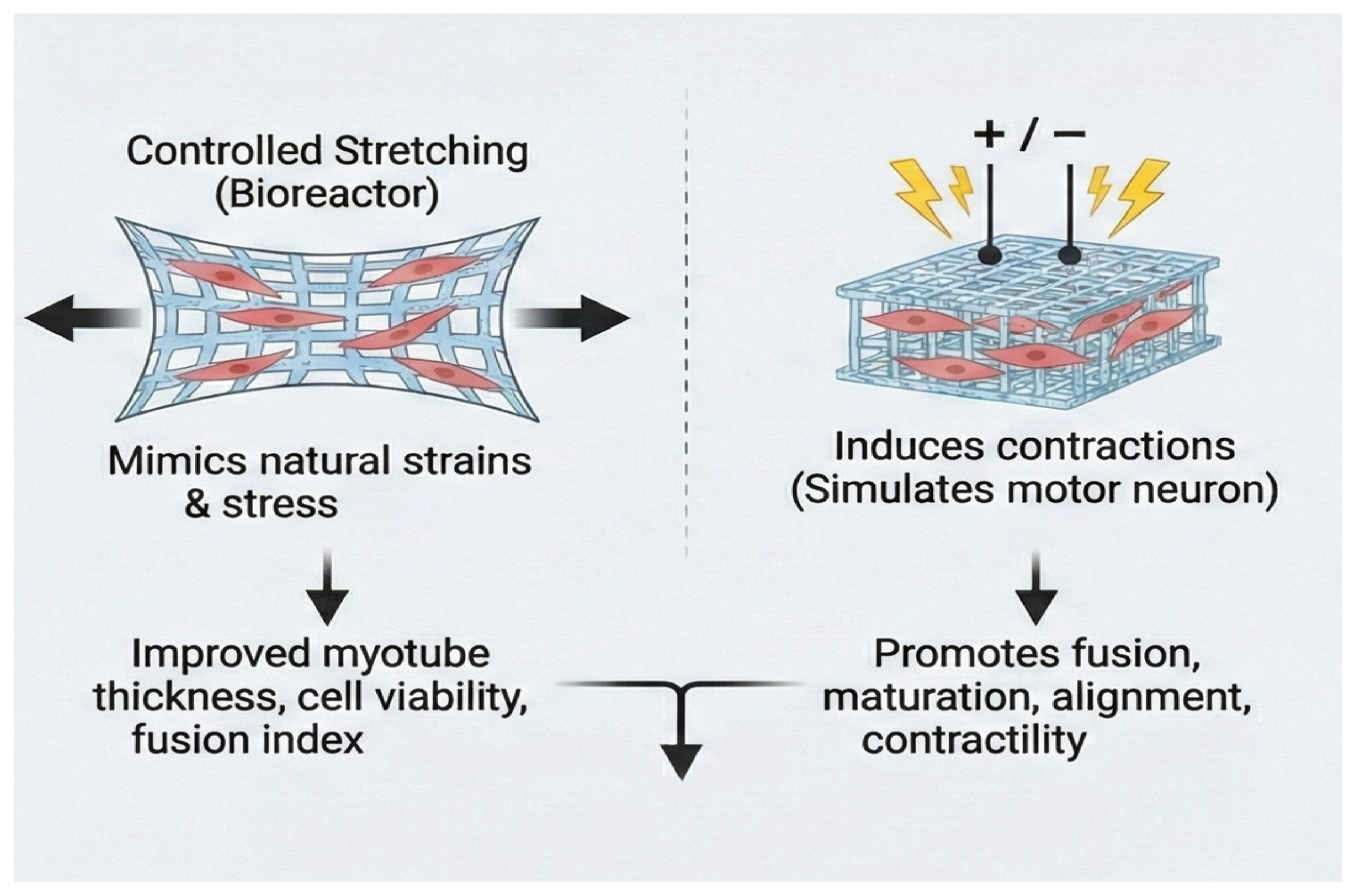
Multi-modal stimulation strategies for enhanced muscle tissue development. “This figure was generated using the AI-assisted design tool FigureLabs. The initial layouts were subsequently refined, labeled, and verified by the authors to ensure scientific accuracy”.

**Figure 11:**
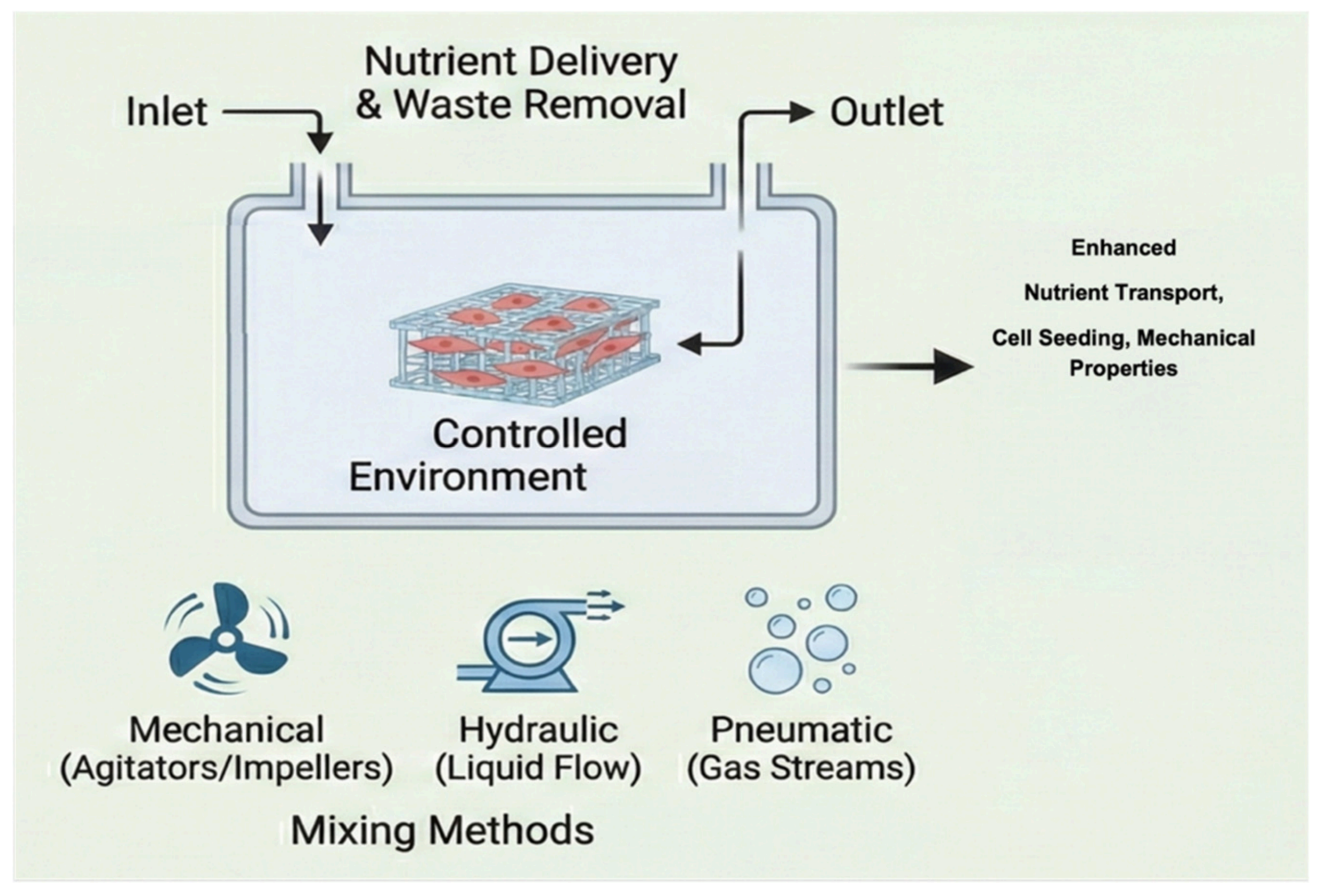
Controlled bioreactor environment for optimized myogenic maturation. “This figure was generated using the AI-assisted design tool FigureLabs. The initial layouts were subsequently refined, labeled, and verified by the authors to ensure scientific accuracy”.

Bioreactors specialized for myocyte maturation are designed to replicate the necessary conditions for development and proliferation in controlled environments. Maturation can be stimulated via application of mechanical and electrical stimulation along with nutrient delivery (Figure 10). Bioreactors can be classified based on mixing methods (Figure 11), for example, mechanical methods use agitators or impellers, hydraulic methods utilize liquid flow, and pneumatic methods employ gas streams for mixing [131]. It is essential to recreate the natural physiological environment of undamaged native muscle tissue. Satellite cells are an abundant stem cell with well-established methods for isolation and in vitro culture, making them a viable choice for differentiation via bioreactor. The stability of bioreactor conditions and a suitable culture media are essential for the maturation and proliferation of competent cells. The most common serum added to culture media is fetal bovine serum but it possesses risks such as contamination. Alternatives have been studied with promising results, such as platelet lysates and disaccharides in protein-free cell cultures [132]. Applying a mechanical force to the nanofiber scaffolds in vitro via stirred flask or direct perfusion directs transport of nutrients and oxygen along with waste removal by convective transport while enhancing cell seeding. Mechanical conditioning such as cyclical mechanical stretching has shown evidence to enhance the mechanical properties of tissues generated by skeletal muscle cells suspended in collagen and improve proliferation and alignment of human heart cells seeded on a scaffold with gelatin-matrix [133,134]. By combining and fine-tuning these characteristics and conditions, bioreactors can improve the efficiency and uniformity of cell seeding onto nanofiber scaffolds.

Coeyman et al. [135] were able to design a novel 3D-printed bioreactor comprising six-well plates with magnetic pistons controlled by an external electromagnet that enabled the team to precisely manipulate the forces applied in stretching of 3D fibrin gel matrix with embedded cardiac fibroblasts. The fibrin gel scaffold was cyclically uniaxially stretched in the bioreactor at an average of ~10% stretch at a frequency of 0.27 hertz for 3 days changing the media daily. Results from the mechanical testing led to increased collagen accumulation and tissue stiffness when comparing the stretched tissues versus the non-stretched tissue. Immunofluorescent analysis was performed on the tissue samples after the final day of stretching, revealing an induced change in cell morphology where stretched cells exhibited greater elongation and alignment compared to non-stretched cells. Although this study does not directly involve skeletal muscle most affected by VML injuries, these principles and insights can greatly contribute to the maturation and proliferation of myocytes.

Cullen and Das [136] fabricated pre-innervated tissue-engineered muscle (INTEM) by growing neuron-myocyte co-culture on electro-spun aligned nanofiber sheets. INTEMs are cultures of mouse skeletal myocytes that are grown in combination with rat spinal motor neurons. A novel bioreactor was utilized to facilitate axonal stretch growth and development of the INTEMs promoting the reinnervation and regeneration of muscles.

After incubating the INTEMs for 7 days the team noted longer and thinner myofibers with an increased myocyte fusion index from neuron-myocyte co-culture that was not present in non-innervated cultures. The INTEM construct was tested using a rat model with VML injury which produced significantly more muscle volume than was recovered after 3 weeks compared to non-innervated constructs. Ultimately the study team noted a significantly higher number of satellite cells and revascularization around the site of injury critical for competent and functional muscle cell recovery along with increased production of mature neuro-muscular junctions [136].

## 7. Vascularization and Innervation: Critical Requirements for Functional Integration

The functional integration of any bioengineered construct into host tissue is dependent on two biological processes that remain the greatest challenges in the field: revascularization, involving the formation of a perfused capillary network within the construct, and reinnervation, involving the establishment of functional NMJs between motor axons and regenerated myofibers [137]. In the absence of vascularization, constructs will become necrotic within days of implantation. In the absence of reinnervation, myofibers fail to generate voluntary contractile force, and will undergo disuse atrophy, regardless of initial myofiber quality. While biomaterial design, cellular composition, and biophysical stimulation can collectively support myogenesis, the long-term survival and functional integration of implanted constructs in vivo is ultimately dependent on the establishment of a perfused vascular network and functional NMJs.

As previously discussed, skeletal muscle is one of the most densely vascularized tissues within the body, with each myofiber lying within 25–30 µm of a capillary in order to support the high metabolic demands of contraction [11,138]. This vascular density must be matched in engineered constructs, particularly for the treatment of VML, where sufficient diffusive transport is critical for tissue repair and regeneration. The vascularization challenge has been approached through three principal strategies: (1) pre-vascularization in vitro, in which endothelial cells and pericytes are co-cultured with myoblasts in the scaffold to form a vascular network prior to implantation, (2) angiogenic growth factor delivery to promote vascularization following implantation, and (3) surgical anastomosis of pre-formed vascular channels with host vessels at the time of implantation.

The pre-vascularization approach exploits the self-organizing capacity of endothelial cells. When co-cultured with pericytes or smooth muscle cells in a three-dimensional environment, human umbilical vein endothelial cells (HUVECs) or human dermal microvascular endothelial cells (HDMECs) form lumenized capillary networks through a process recapitulating vasculogenesis [139]. Lesman et al. demonstrated that the implantation of PLLA/PLGA sponge scaffolds pre-vascularized with endothelial cells (i.e., HUVECs), fibroblasts, and myoblasts resulted in vessel-like network formation with increased perfusability [138]. Additionally, Czajka et al. demonstrated that the implantation of pre-vascularized constructs into hindlimb defects in mice led to anastomosis with host capillaries within three days as well as increased expression of Pax7 and MyoD, suggesting that satellite cells were able to migrate to the injury site [140]. Das et al. demonstrated that pre-innervated myocytes co-cultured with spinal motor neurons (INTEMs) subjected to axonal stretch growth through a bioreactor produced myocytes with increased satellite-cell proliferation, fusion indices, muscle volume, and chiefly, revascularization around the injury site [136]. Furthermore, pre-vascularized scaffold constructs enable effective diffusive drug transport, allowing for the delivery of therapeutic agents to the injury site. Madden et al. demonstrated that the administration of pharmacological agents to pre-vascularized myobundles led to myofiber hypertrophy, which paralleled the effects of agents on native muscle tissue [141].

The angiogenic growth factor delivery approach aims to stimulate host endothelial cell proliferation and migration into the scaffold. As previously discussed, the delivery of growth factors (i.e., VEGF, FGF, PDGF) has been shown to augment muscle hypertrophy; however, the primary limitation is the lack of mechanical integrity reproduced in existing muscle, which can be resolved with functionalization of growth factors onto scaffold constructs [35]. Zhang et al. reported that the dual delivery of VEGF-A and PDGF-BB within a chitosan hydrogel with PEG/PLCL electrospun fibers synergistically promoted revascularization when implanted in the carotid artery of rabbits. However, the concentration and timing of growth factor delivery must be carefully controlled; persistent supraphysiologic VEGF produces immature, leaky capillaries, while physiologic gradients drive the formation of mature, perfused capillaries [142]. Notably, while the release of PDGF promoted vascular smooth muscle cell proliferation, the release of VEGF inhibited fast proliferation of cells at the beginning, preventing mitogen-induced hyperplasia [143].

The surgical anastomosis approach includes the arteriovenous (AV) loop model, in which a vascular loop formed from a donor vein graft is placed within a protection chamber containing the scaffold construct, allowing neovascular outgrowth from the loop into the construct, after which it is transposed to the injury site [144]. Dolderer et al. demonstrated that the implantation of vascularized adipose tissue within PLGA sponge scaffolds into rats with tissue defects resulted in a significant increase in revascularization, with no observable fat necrosis [145]. The application of surgical anastomosis to muscle constructs is demanding and introduces an additional surgical procedure, although it may be the only viable approach for large defects (>50 cm^3^) that cannot be adequately perfused by vascular ingrowth approaches alone.

The innervation of scaffold constructs is a later-stage challenge that has received far less attention than vascularization despite being equally critical for functional contractile force generation. The process of reinnervation involves motor neurons, which must extend axons from the closest intact nerve stump across a centimeter-scale through the construct to establish NMJs on individual myofibers at a density equal to native innervation, anywhere from 1:10 to 1:2000 motor neurons to myofibers depending on whether the muscle requires precise control or gross force. Unfortunately, this process takes weeks to months, even in the most favorable in vivo environments, which requires that constructs maintain long-term integrity and viability [146].

The reinnervation challenge has been approached through four principal strategies: (1) embedding neural guidance channels within the scaffold that align axon growth toward the construct, (2) delivering neurotrophic growth factors (e.g., BDNF, GDNF, neurotrophin-3) to establish gradients that attract existing axons, (3) co-implanting induced motor neurons or neural progenitor cells derived from iPSCs, and (4) creating pre-fabricated microfluidic devices with myotubes and motor neurons that can be implanted as integrated neuromuscular units [147,148,149,150,151,152]. Despite their promise, these approaches are limited by issues with immunological complexity, scalability, and long-term integration.

The integration of vascularization and innervation strategies within a single scaffold platform has only occasionally been attempted, even though these processes are spatially and temporally coupled; blood vessels and nerves travel together in neurovascular bundles and share signaling pathways. The development of multi-factor delivery systems within scaffold constructs that present vasculotrophic cues in central channel domains and neurotrophic cues in peripheral channel domains represents a promising but underexplored design concept. Ultimately, achieving revascularization and reinnervation while maintaining the myogenic architecture of the scaffold represents the greatest current design challenge in the field.

## 8. Immune Modulation as a Therapeutic Strategy in VML

The immune response to skeletal muscle injury extends beyond a passive pathological role; it plays a central regulatory role in the regenerative process. A coordinated, time-dependent sequence of innate and adaptive immune responses beginning with neutrophil infiltration, progressing through pro-inflammatory M1 macrophage activations and anti-inflammatory M2 macrophage predominance, and resolving with T-cell modulation and extracellular matrix (ECM) remodeling is required for effective muscle repair [153].

Classically activated M1 macrophages, induced by IFN-γ and TNF- α, phagocytose debris and produce pro-inflammatory cytokines, including IL-1β, IL-6, and IL-12, that prepare the wound, but can inhibit myoblast differentiation if sustained. Alternatively activated macrophages, induced by IL-4, IL-10, and IL-13, promote satellite-cell proliferation, ECM remodeling, and angiogenesis through secretion of IGF-1, TGF- β1, and VEGF [154]. Liu et al. demonstrated that the depletion of M2 macrophages severely impaired myofiber formation and regeneration [155].

Biomaterial scaffolds can be designed to actively direct macrophage polarization toward a pro-regenerative M2 phenotype. Sicari et al. demonstrated that implantation of ECM-based scaffolds in VML models promoted M2 macrophage polarization rather than domination of M1 macrophage activation that is typically observed with synthetic material scaffolds, thus facilitating myogenesis [156]. ECM scaffolds designed to retain specific proteins, including fibronectin, laminin, and collagen IV, as well as designed to release specific cytokines, including IL-4 and IL-33, have been shown to drive M2 polarization, thus enhancing satellite-cell-mediated regeneration in VML [157]. The integration of immune modulation with biomaterial scaffold platforms represents a systems-level approach to VML that addresses the upstream immunological barriers to downstream muscle repair.

## 9. Regulatory Consideration and FDA Approval Pathways for VML Scaffold Platform Therapeutics

Current clinical treatments remain limited for VML [158,159]. The standard approach, free functional muscle transfer (FFMT), involves transplanting muscle tissue with its associated nerves and vasculature from another part of the body. Although FFMT can partially restore function, it is associated with significant donor-site morbidity, long surgical procedures, limited tissue availability, and unpredictable functional recovery [6,160]. At present, there are no FDA-approved regenerative products specifically for treating VML [161]. Still, several regenerative biomaterials and extracellular matrix (ECM) scaffolds, such as decellularized dermal matrices (AlloDerm®, LifeCell Corporation/AbbVie (Allergan Aesthetics), Branchburg, NJ, USA), porcine small intestinal submucosa (OASIS®, Cook Biotech, Inc., West Lafayette, IN, USA), urinary bladder matrix (MatriStem® ACell, Inc. (acquired by Integra LifeSciences), Columbia, MD, USA and Lafayette, IN, USA), and Allogeneic Cultured Keratinocytes and Fibroblasts in Bovine Collagen (GINTUIT®, Organogenesis Inc., Canton, MA, USA), have received FDA clearance for soft-tissue reconstruction and wound repair. This shows that biologically derived scaffold platforms can meet regulatory standards. Although these products are not approved for VML, they set important examples for bringing new muscle regenerative scaffolds into clinical use.

The successful clinical translation of emerging VML therapies requires careful alignment with the regulatory framework governing advanced therapeutic products in the United States. The Food and Drug Administration (FDA) regulates potential scaffold platform therapeutics under several overlapping regulatory categories depending on their composition and mechanism of action, including medical devices, biological products, drugs, and combinations thereof. Bringing products from the lab to the clinic is not just about meeting regulations, it is also about making sure every batch is safe and works the same way. When scaffolds contain living cells, exosomes, or growth factors, they must be manufactured under current good manufacturing practice (cGMP) to remain sterile and reliable. To get approved and actually used, manufacturers need clear quality checks, proven methods, and set standards for when the product is good to go.

Understanding this framework is essential for investigators designing preclinical and clinical programs, as regulatory pathway selection profoundly influences the nature and extent of evidence required for market approval [162].

Acellular biological scaffolds derived from decellularized ECM occupy a complex regulatory position that depends on their processing, components, and intended use. Products that are minimally manipulated, composed solely of structural ECM components, and implanted at homologous anatomical sites may qualify for the Human Cells, Tissues, and Cellular and Tissue-Based Products (HCT/P) qualification pathway under 21 CFR Part 1271, which requires registration and listing, but not Premarket Approval based on 21 CFR Part 1271. Human Cells, Tissues, and Cellular and Tissue-Based Products. Code of Federal Regulations. However, scaffolds that are more than minimally manipulated, for example, crosslinked or combined with synthetic polymers, or loaded with biological material, lose HCT/P eligibility and fall under stricter Premarket Approval (PMA) requirements as devices, or as biologics regulated under the Public Health Service Act based on the FDA. Guidance for Industry and FDA Staff: Preparation of IDEs and INDs for Products Intended to Repair or Replace Knee Cartilage. 2011.

Biological scaffolds combining scaffold with living cells or drugs fall under the category of combination products, which are among the most complex regulatory designations. The FDA determines the primary mode of action of combination products and assigns review authorities, including the Center for Devices and Radiological Health (CDRH), Center for Biologics Evaluation and Research (CBER), or Center for Drug Evaluation and Research (CDER). This designation requires comprehensive characterization of the cellular component, demonstration of sterility, and evidence of effectiveness. The Regenerative Medicine Advanced Therapy (RMAT) guidelines provide specific regulatory direction for scaffold platforms. Expedited Programs for Regenerative Medicine Therapies for Serious Conditions [163].

**Figure 12:**
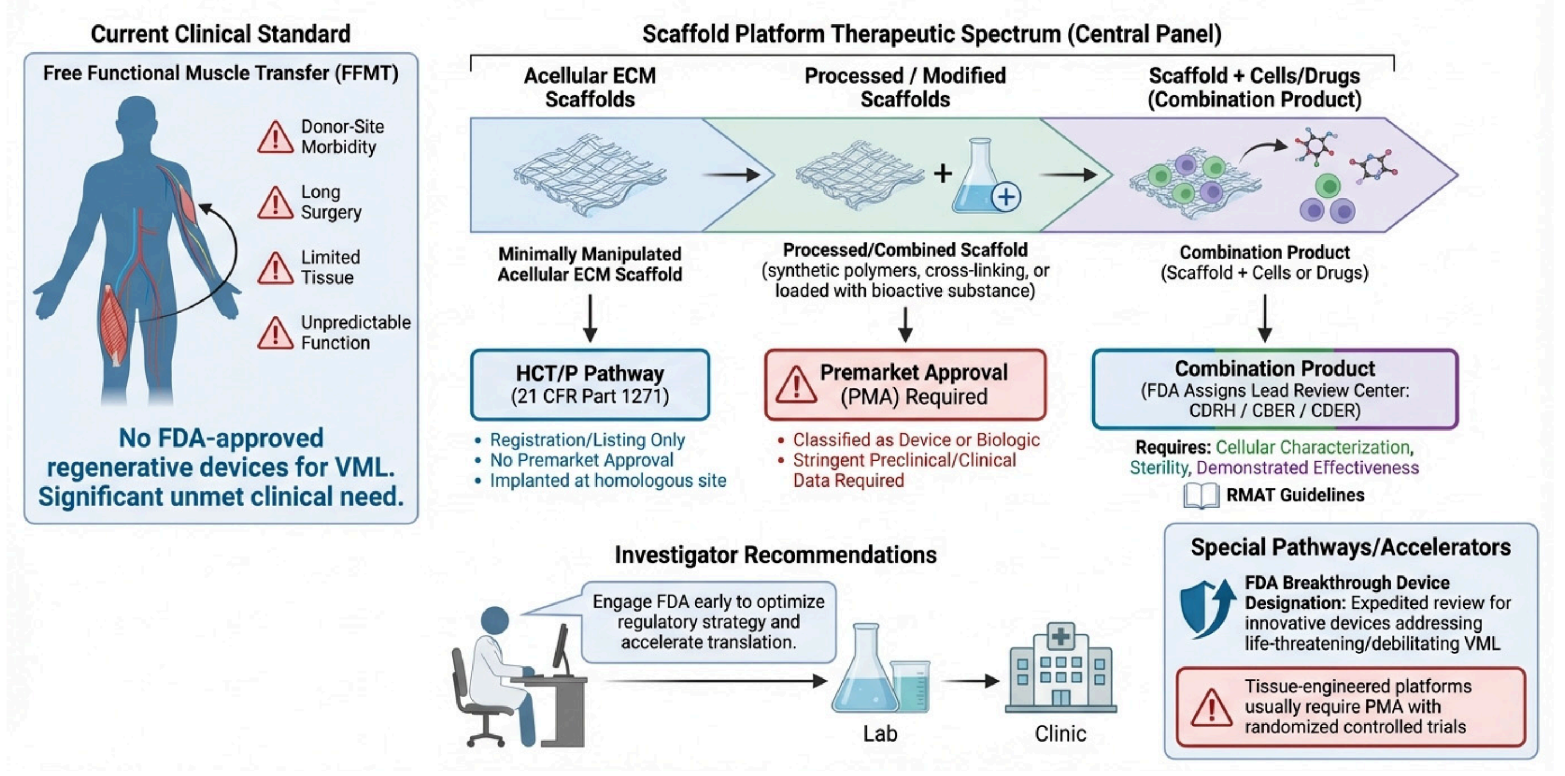
Regulatory landscape for scaffold-based therapies in volumetric muscle loss (VML), illustrating the transition from current surgical treatments to advanced multifunctional regenerative scaffold platforms and associated FDA pathways. “This figure was generated using the AI-assisted design tool FigureLabs. The initial layouts were subsequently refined, labeled, and verified by the authors to ensure scientific accuracy”.

Tissue-engineered scaffold platforms intended for VML treatment that involve substantial manufacturing complexity will likely require the PMA pathway, which demands the highest level of evidence for safety and effectiveness involving randomized controlled clinical trials. Additionally, tissue-engineered scaffold platforms may be eligible for designation through the FDA’s Breakthrough Device Designation program, which offers an expedited pathway for innovative medical devices that provide effective treatment for life-threatening or irreversibly debilitating conditions [164]. Ultimately, researchers pursuing scaffold platform therapies for VML should engage with the FDA at the earliest feasible stage to align on regulatory strategy, minimize duplication of effort, and accelerate the translation of laboratory advances to clinical benefit (Figure 12).

## 10. Conclusions

VML is a fundamentally unresolved clinical problem, mainly due to the difficulty of coordinating the complex, multiscale processes required for functional muscle regeneration rather than a lack of individual therapeutic components. Effective repair requires the concurrent restoration of vascularization, innervation, immune balance, and contractile architecture. These processes are highly interconnected and subject to dynamic regulation.

Current research is shifting from reductionist approaches to integrated, systems-level strategies that rationally combine biomaterials, cells, and bioactive signals. Scaffold platforms are advancing from passive structural supports to dynamic systems that modulate cellular behavior, immune responses, and tissue remodeling. Design parameters, including microarchitecture, mechanical properties, degradation kinetics, and bioactivity, must be precisely engineered to correspond with key biological processes such as satellite-cell activation, macrophage polarization, angiogenesis, and neuromuscular junction formation.

Emerging technologies, including 3D bioprinting, nanofiber engineering, exosome-functionalized scaffolds, and bioreactor-driven maturation, offer extraordinary control over the spatial and temporal orchestration of regenerative cues. Successful clinical translation of these inventions will require not just advances in biology and engineering but also early and tactical alignment with regulatory systems established by the U.S. Food and Drug Administration. Most next-generation volumetric muscle loss therapies are expected to be classified as combination products, necessitating integrated evaluation of device performance, biological activity, manufacturing reproducibility, and long-term safety.

Major challenges for clinical translation include achieving scalable vascularization and stable long-term innervation, establishing standardized and good manufacturing practice (GMP)-compliant production processes, defining robust potency and quality metrics for complex constructs, and displaying consistent functional recovery in large-animal and human studies. Early engagement with regulatory agencies and integration of regulatory considerations during the design phase are vital to mitigate development risks and expedite medical adoption.

Future research should prioritize the development of integrated regenerative platforms capable of simultaneously restoring skeletal muscle fibers, vascular networks, motor innervation, extracellular matrix organization, and immune homeostasis, rather than optimizing these components in isolation. Promising approaches include multifunctional scaffold systems that combine biomaterials with controlled delivery of exosomes or other bioactive molecules, advanced biofabrication technologies such as three-dimensional bioprinting and electrospinning, and bioreactor-mediated tissue maturation to replicate the dynamic regenerative microenvironment of native skeletal muscle. Additionally, the establishment of standardized preclinical models, clinically relevant functional outcome measures, and reproducible manufacturing processes is essential to facilitate cross-study comparisons and accelerate clinical translation.

Moving forward, progress in treating VML will rely on developing regenerative platforms that combine biomaterials, bioactive signals, new biofabrication methods, and controlled physical stimulation, all within frameworks ready for clinical use and regulation. While preclinical studies have made significant strides, most of the regenerative approaches covered here are still experimental and need more testing in large-animal models and well-designed clinical trials. Ongoing teamwork among bioengineers, clinicians, regulatory experts, and industry partners will be key to solving the remaining scientific and practical challenges. As these efforts continue, integrated regenerative strategies could greatly improve muscle repair and help patients with volumetric muscle loss achieve better outcomes and quality of life.

## Data Availability

The original contributions presented in this study are included in the article. Further inquiries can be directed to the corresponding authors.

## Abbreviations

The following abbreviations are used in this manuscript:

VML	volumetric muscle loss
ECM	extracellular matrix
FDA	Food and Drug Administration
μm	micrometer
MRFs	myogenic regulatory factors
bHLH	Basic Helix-Loop-Helix
MyoD	myogenic differentiation 1
DMD	Duchenne Muscular Dystrophy
FFMT	free functional muscle transfer
GelMA	gelatin methacrylate
RGD	arginylglycylaspartic Acid
CNTs	carbon nanotubes
PPY	polypyrrole
PANI	polyaniline
ES	electrospinning
iPSCs	Induced Pluripotent Stem Cells
hESCs	human embryonic stem cells
NMJ	neuromuscular junction
MSCs	mesenchymal stem cells
ADSCs	adipose-derived stem cells
MPC-Exo	Myogenic Progenitor Cell-Derived Exosomes
qRT-PCR	Quantitative Reverse Transcription Polymerase Chain Reaction
MYOD	Myogenic Differentiation Marker
MYOG	myogenin
PAX7	Paired Box 7
CCK-8	Cell Counting Kit-8
SD	Standard Deviation
TNF-α	tumor necrosis factor-alpha
NF-κB	Nuclear Factor Kappa B
PRP	platelet-rich plasma
α-SMA	Alpha-Smooth Muscle Actin
VEGF	vascular endothelial growth factor
PI3K/Akt	Phosphoinositide 3-Kinase/Protein Kinase B
sEVs	small extracellular vesicles
ATP	Adenosine Triphosphate
PGC-1α	Peroxisome Proliferator-Activated Receptor Gamma Coactivator 1-Alpha
IL	Interleukin
Hz	hertz
INTEM	innervated tissue-engineered muscle
NT-3	neurotrophin-3
BDNF	Brain-Derived Neurotrophic Factor
AChRs	Acetylcholine Receptors
IFN-γ	Interferon-Gamma
IGF-1	insulin-like growth factor-1
TGF-β1	transforming growth factor-beta 1
HCT/P	Human Cells, Tissues, and Cellular and Tissue-Based Products
PMA	Premarket Approval
CDRH	Center for Devices and Radiological Health
CBER	Center for Biologics Evaluation and Research
CDER	Center for Drug Evaluation and Research
RMAT	Regenerative Medicine Advanced Therapy
GMP	good manufacturing practice
